# Guideline-based strategies to identify severe cytokine release syndrome in COVID-19 and cancer immunotherapy using large-scale electronic health records

**DOI:** 10.3389/fdgth.2025.1625889

**Published:** 2026-02-17

**Authors:** Philippe A. Robert, Jonas Denck, Cao Tri Do, Elif Ozkirimli, Candice Jamois, Chiara Corso, Ken Wang, Christoph T. Berger

**Affiliations:** 1 Roche Pharmaceutical Research and Early Development, Roche Innovation Center, F. Hoffmann-La Roche AG, Basel, Switzerland; 2 Translational Immunology, Department of Biomedicine, University of Basel, Basel, Switzerland; 3 Roche Global Informatics, F. Hoffmann-La Roche AG, Kaiseraugst, Switzerland; 4 Roche Information Solutions, F. Hoffmann-La Roche, Basel AG, Switzerland; 5 Roche Product Development, F. Hoffmann-La Roche, Basel AG, Switzerland; 6 University Center for Immunology, University Hospital Basel, Basel, Switzerland

**Keywords:** Electronic Health Records, Optum®, COVID-19, T-cell engager (TCE), cytokine release syndrome (CRS), ASTCT grading, case identification

## Abstract

**Introduction:**

Cytokine Release Syndrome (CRS) is a life-threatening adverse event of cancer immunotherapies and a complication of infections. Predicting which patients are at risk for severe CRS would inform mitigation decisions and drug development, but requires large, reliably labeled datasets.

**Methods:**

This study evaluates the feasibility of disease-agnostic case identification of CRS patterns in large-scale Electronic Health Records (EHR) to generate high-quality cohorts of CRS-positive and CRS-negative patients.

**Results:**

Using the Optum® de-identified COVID-19 EHR dataset, we isolated 2.5 million patients with active COVID-19 and 171 individuals treated with the T-cell Engager (TCE) blinatumomab. Diagnosis codes for CRS were underutilized and provided limited information on severity. Instead, we implemented the consensus CRS grading guidelines, which identified 92,541 COVID-19 patients (3.7%) and 54 blinatumomab patients (31.5%) with grade 2 or higher CRS, respectively. Severe CRS COVID-19 patients showed heterogeneous inflammatory levels.

**Discussion:**

Our EHR-based CRS case identification strategy is suitable for risk factor analysis and developing CRS risk prediction models.

## Highlights

Medical ICD CRS codes are reported inconsistently in EHR datasets resulting in a continued need for clear and reproducible definitions of severe CRS across various EHR datasets and conditions.
Identification of severe CRS is possible by implementing accepted guidelines based on individual patient trajectories.
CRS grades in TCE patients are robust to uncertainties from the guidelines, whereas COVID-19 patients exhibit heterogeneity in the degree of symptom severity and inflammation.
We propose combining inflammatory and grading criteria in COVID-19 patients to distinguish between actual hyperinflammatory CRS patients and those fulfilling some CRS criteria, such as those with lung damage without hyperinflammation.

## Introduction

Cytokine Release Syndrome (CRS) is a life-threatening adverse event of cancer immunotherapies and a complication of infections such as COVID-19, malaria or dengue (1, 2). CRS is characterized by an excessive immune response with high systemic levels of pro-inflammatory cytokines, which in turn induce cytokine-mediated vascular leakage causing hypotension and pulmonary edema resulting in hypoxia and potentially multi-organ failure or neurotoxicity (3).

Severe COVID-19 infection, responsible for the overload of healthcare systems during the early phases of the COVID-19 pandemic, has been associated with CRS, marked by high levels of systemic inflammation, including CRP, ferritin, and IL-6 (4). Persistent innate activation has been discussed as the root cause of CRS in COVID-19 (5). Anti-inflammatory and cytokine-blocking strategies have been successfully employed to treat severe COVID-19 (6, 7). In the context of immune-activating agents, such as chimeric antigen receptor (CAR-T) cells or T-cell engager bispecific antibodies (8), up to 46% of patients experience severe CRS (9), depending on the treatment and dose. Current mitigation strategies, which include vasopressors, ventilation support, cytokine-blocking therapies, and corticosteroids, show variable success across patients (9). Further, despite refined dose-escalation strategies for new drugs or preemptive anti-inflammatory treatments, several clinical studies are still terminated due to fatal CRS events. Thus, CRS represents a significant challenge in the development and safe administration of new cancer immunotherapies and remains a high risk for patients.

The reasons why some patients develop CRS while others do not remain unclear, and risk factors for CRS are still unknown. For specific drugs, baseline target abundance or tumor burden, as well as drug dose, have been linked with higher incidence of severe CRS (10–12), but it is unclear if these findings translate between drugs and conditions. In order to understand which potential risk factors are common features of severe CRS across conditions, it is of critical interest to analyze which patients developed CRS across large datasets, including different triggers such as infection and TCE therapy. Furthermore, narrowing down which patient features are linked with severe CRS occurrence will ultimately allow the development of predictive models assessing for severe CRS risk prediction (10), which are currently limited to a very small application range, datasets and feature space (11, 13). Robust predictive models would facilitate the forecasting of at-risk patients to be considered for tailored monitoring (e.g., hospitalization) or mitigation (e.g., step-up dosing regimen or selected anti-inflammatory drugs). Additionally, it would facilitate a more accurate evaluation of the patient's individualized risk-benefit profile for a compound being developed in a target population. Building predictive models is only possible with large and diverse datasets that contain reliably labeled patients experiencing severe CRS, and cover, to a certain extent, the potential variability in CRS profiles and patient comorbidities.

Large-scale electronic healthcare records (EHR) offer new opportunities to study real-world patient heterogeneity, rare conditions and comorbidities (14, 15). They allow to extract insights into risk factors beyond clinical trial data, which are limited in size and availability of prior patient history. By analyzing EHR datasets, we can investigate commonalities in patient dynamics, risk factors, and mitigation responses, between various CRS-inducing conditions, potentially identifying predictive features transferable across conditions.

Extracting reliable information from EHR datasets can be challenging due to their high dimensionality in medical codes and a high level of noise (16). Additionally, it is difficult to control how information was reported, when lab values were taken, and there may be inconsistencies in the recording of patients’ complete medical histories. For instance, medical diagnosis codes for CRS have only recently been implemented in EHR data (i.e., earlier CRS patients may not be detectable) and they do not report the criteria used for the grading. Therefore, to effectively utilize EHR datasets, a disease-agnostic, evidence-based CRS case identification strategy is required that is robust to heterogeneous reporting strategies and potential missing data.

In immuno-oncology, CRS grading systems such as the American Society for Transplantation and Cellular Therapy (ASTCT) consensus (17) have been developed. Five grades have been proposed, starting with isolated fever without other identifiable cause for grade 1, escalating to grades 2–4 depending on the presence of hypotension or hypoxia and on the need for non-invasive/invasive ventilation or vasopressor support. Grade 5 represents fatal CRS. The ASTCT consensus was designed to support fast mitigation decisions towards life-threatening symptoms. It deliberately excluded markers of systemic inflammation because commonly used markers, such as C-reactive protein (CRP) only become detectable in the blood after a delay of up to 24 h and early biomarkers such as cytokines are more seldom measured. In infectious diseases such as COVID-19, ASTCT-like CRS gradings have not been widely used because infection-mediated lung injury may contribute to hypoxia or respiratory failure without hyperinflammatory grounds and confound the grading. Several CRS inflammatory scores (18) have been proposed for COVID-19. They are based on inflammatory markers, blood biochemical analysis, evidence for organ failure or lymphocytopenia, such as the c-HIS (19), Cov-Hi (20), Temple (21), or, most recently, one that included mechanical ventilation as a criterion (22). While those inflammatory scores were typically developed to predict fatal outcomes, their correlation with CRS symptoms and grading is, therefore, uncertain. To ensure transferability of findings between infection- and immunotherapy- induced CRS, it is important to use the same definition of CRS across the studied conditions and to understand the overlap and limitations between different case identification algorithms.

In this study, we explore the feasibility of reliably identifying patients with different severity of CRS in EHR data across diseases and datasets using different identification strategies. Specifically, we used (i) the International Classification of Disease (ICD) codes for CRS, (ii) the ASTCT grading system (17) and (iii) inflammatory CRS definitions (19, 20, 22) to identify severe CRS cases. The ICD codes serve as a reference of clinician labelled CRS with the risk of underreporting, the ASTCT serve as an objective symptom-based and widely-used standard which has not yet been applied in COVID-19 context, and the inflammatory CRS definitions serve as a more mechanistic grading system that has not yet been used in onco-immunology. We assess the overlap between these criteria, and propose definitions of positive and negative cohorts for high-grade (i.e., grade 2+ or 3+) CRS in COVID-19 or immunooncology.

Using the Optum® de-identified COVID-19 Electronic Health Record data set (‘Optum® COVID-19 data’), we compared the occurrence of severe CRS following those three case definition strategies on 2.5 million patients with COVID-19 infection and 171 patients treated with the TCE blinatumomab. We find that ICD medical codes for CRS are not directly suitable to detect all CRS cases. We propose an algorithmic implementation of the ASTCT grading and inflammatory criteria applicable to any EHR dataset. Finally, we observe a high heterogeneity of inflammatory profiles in COVID-19 patients, suggesting the existence of patient subsets with potentially different individualized responses to mitigation. Our findings demonstrate the feasibility of CRS case identification in EHR datasets, which is the first necessary step before analysis of CRS risk factors, potential stratification of CRS patients and the ultimate development of individualised machine learning-based CRS risk prediction models.

## Results

### Cohort identification workflow and patient characteristics

**Figure 1:**
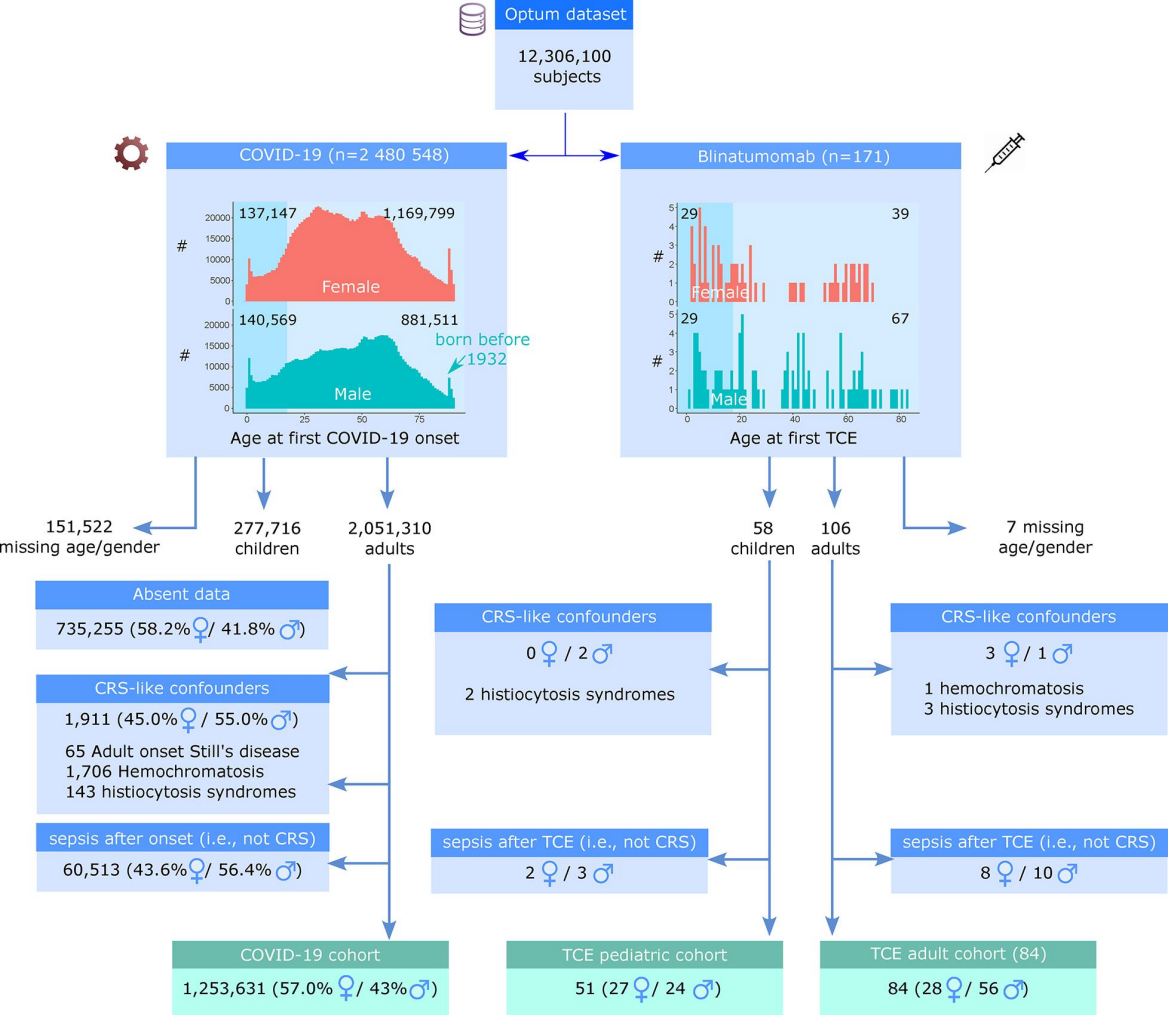
Definition of the cohorts of interests, inclusion and exclusion criteria. From the Optum® COVID-19 data, subjects with active COVID-19 or who received TCE treatment (blinatumomab) were isolated. The first COVID-19 infection or TCE treatment was considered a triggering event. Subjects with missing information on gender or age were excluded. Subjects with pre-existing comorbidities that share traits with CRS at least 7 days before the triggering event were excluded. A significant number of COVID-19 patients had no reported data 30 days around the triggering event and were excluded. Patients diagnosed with Sepsis within 30 days after onset were also excluded. Three cohorts of interest were used for subsequent CRS case identification: ‘COVID-19 adult cohort’, ‘TCE adult cohort’ and ‘TCE pediatric cohort’.

The Optum® COVID-19 data contains longitudinal EHR of 12 million U.S. patients, with annotated demographics, lab values, ICD codes for procedures, diagnostics, and medication. We isolated two cohorts to test whether we can reliably identify CRS patients (Figure 1). The COVID-19 cohort contained 2.5 million subjects with active COVID-19 infection, as detected based on a positive antigen or PCR tests or a COVID-19 ICD-10 diagnosis code (see Methods). The second cohort consisted of 171 subjects on blinatumomab TCE treatment regardless of COVID-19 infection. The first active COVID-19 diagnosis or the first blinatumomab infusion was defined as the triggering event time for each cohort, respectively.

The demography of the two cohorts is summarized in Figure 1. We identified 2,051,310 adult (i.e., 18 years or older) COVID-19 patients with known demographics. For the TCE cohort, we kept both adult and pediatric patients as two separate sub-cohorts. To reduce confounders, we used ICD diagnosis codes to exclude patients with pre-existing comorbidities that could potentially mimic CRS, such as autoinflammatory disease. Given that patients with sepsis may exhibit identical clinical findings or laboratory values as those with CRS, we next excluded 60,513 adult COVID-19 and 23 TCE patients based on a sepsis diagnosis code within 30 days after the triggering event. An additional 735,000 adult COVID-19 patients were excluded from further analysis because of absent clinical data, except for the positive COVID-19 test. The remaining 1.25 million adult COVID-19 patients were included in the further analysis. We kept a cohort of 84 adult and 51 pediatric TCE patients to be assessed for CRS. The COVID-19 patients were predominantly women (714,462; 57.0%); the adult TCE cohort contained 28 women and 56 men, and the pediatric TCE cohort 27 girls and 24 boys.

### Graded CRS ICD codes are under-reported in the Optum® COVID-19 data

CRS was introduced into international coding systems in January 2020 as an ICD-10 code, with (D89.831 to D89.835) or without (D89.83 or D89.839) information on the grade. The first reported CRS ICD code was dated from August 2020 in our cohorts. We identified 1,194, 11, and 12 patients coded as CRS in the COVID-19, adult TCE, and pediatric TCE cohorts, respectively. Of those, only 94, 9, and 8 were assigned a grade. Data on intra-individual longitudinal changes in grades appeared unreliable because patients were only annotated with the same grade repeatedly. A CRS grade 2 or higher (Grade 2+) code was reported in 56 of 1,253,631 (0.0044%) COVID-19 patients and in 7 of 84 (8.3%, or 6.8% if including sepsis patients or 3.7% from the whole cohort) adult and 3 of 51 (5.9% or 5.3% if including sepsis patients) pediatric TCE patients (Supplementary File 1). Notably, the incidence of CRS in blinatumomab-treated patients in clinical trials is substantially higher (i.e., 10%–15%) (23), and as a rough estimate, COVID-19-related hospitalization rates among unvaccinated infected individuals were approximately 9% (24, 25). Therefore, our results suggest that medical coding for CRS diagnosis was incomplete in the EHR dataset and cannot be used directly to identify CRS patients with high sensitivity.

### Practical implementation of CRS consensus grading on EHR datasets

**Figure 2:**
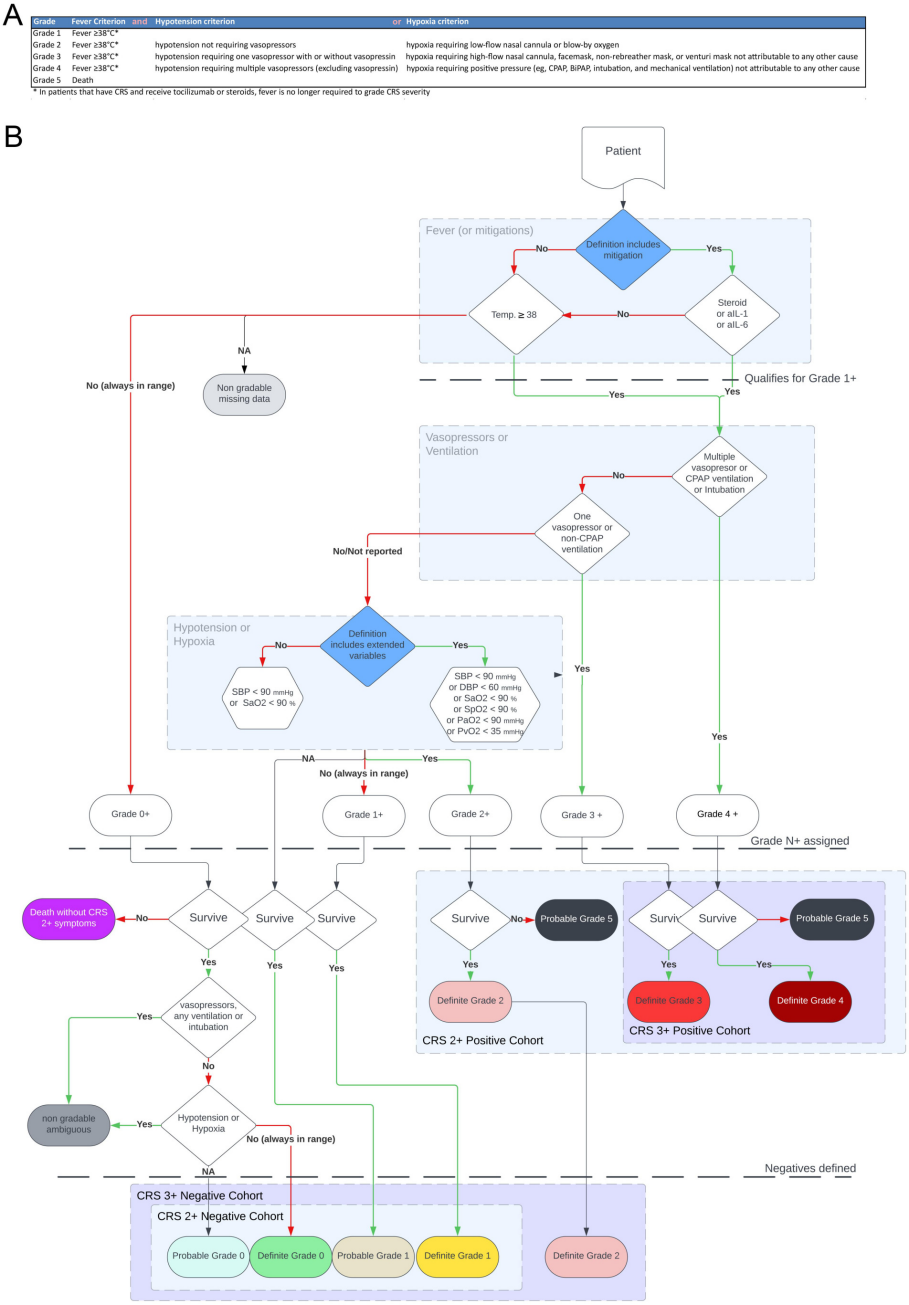
CRS grading algorithm (decision tree) on EHR datasets following the ASTCT grading guideline. **(A)** The latest and commonly used ASTCT CRS grading (17), keeping identical wording as in the original publication. **(B)** From the list of reported patient features within a time window of 30 days after the triggering event (TCE administration or COVID-19 diagnosis), patients are first graded into ‘grade N+’ and then separated into definite grades: grade 1+ includes patients with fever ≥38 °C (‘strict’ definition) or those with potentially mitigated fever by corticosteroid or cytokine blocker (anti-IL1 or anti-IL6) therapy (‘mitigated’ definition). Grade 2+ to 4+ are defined based on the grade-defining interventions: grade 4+: CPAP or invasive ventilation or use of multiple vasopressors; grade 3+ (one vasopressor or non-CPAP ventilation); and grade 2+ (evidence for hypoxia or hypotension). Notably, we assumed that the use of vasopressors or ventilation indicated hypoxia or hypotension, even if the reported cardiovascular or respiratory parameters were within the reference range. Patients without grade 2+ were classified as “definite” grades if lab values were in range, “probable” grades if hypoxia or hypotension were not measured, or as non-classifiable. We proposed a definition for CRS grade 2+ (or grade 3+) positive and negative (i.e., control) cohorts. SaO2 = arterial oxygen saturation, SBP = systolic blood pressure SpO2 = peripheral oxygen saturation, PaO2 = partial arterial oxygen pressure, PvO2 = venous oxygen tension, and DBP = diastolic blood pressure.

Given the limitations for CRS case identification based on ICD codes, we next followed a strategy to identify and grade CRS based on the ASTCT CRS grading guideline (17) (Figure 2A). A key challenge for CRS identification was to match and extract the items needed for ASTCT-based grading to the way patient features were reported in the EHR dataset. We devised an explorative data cleaning strategy linking medical codes for diagnoses, medications, lab values and interventions (procedures) mapping to relevant patient features for CRS (see Methods, Supplementary File 2) and propose a decision tree for CRS grading (Figure 2B), allowing to assign patients to individual grades, as well as ‘grade 2+’ or ‘grade 3+’ CRS positive or negative cohorts. Assigning patients to grade 1+ (i.e., fever +/- other CRS features) or grade 4+ (i.e., requiring multiple vasopressors and positive pressure ventilation) is also feasible but less clinically relevant. We used a time-window of 30 days after COVID-19 infection or TCE administration to extract features of CRS.

Since the ASTCT CRS grading guideline does not specify thresholds to define hypotension nor which markers to use to define hypoxia, we applied thresholds from common clinical practice (26) and proposed three implementations of the guideline (Figure 2B, dark blue nodes). For the most ’strict’ definition of CRS, detection of a fever (≥38 °C) is mandatory, and a single readout, i.e., arterial oxygen saturation (SaO2) and systolic blood pressure (SBP), defines hypoxia or hypotension, respectively. For a second, broader implementation, we allowed an ‘extended definition’ of CRS that additionally considers various hemodynamic parameters and readouts for oxygenation commonly reported in the EHR and in clinical practice. Finally, according to the guideline, patients without fever, but who are receiving fever/inflammation mitigation therapy with glucocorticoid, anti-IL6, or anti-IL1 were included in the ‘extended+mitigation’ definition (17). Assigning grade 5 (i.e., death associated with CRS) was challenging since the EHR dataset reported the time of death, but not the cause of death. To overcome this limitation, we proposed to label deceased patients with evidence of grade 2+ (or grade 3+) CRS prior to death as ‘probable grade 5 CRS’. The remaining deceased patients were labelled as dead without severe CRS symptoms.

To study risk factors for CRS in COVID-19 or TCE therapies, both a positive and a negative cohort for severe CRS are required. The negative cohort should be a well-defined group of patients with the same conditions but who experienced no higher-grade CRS (i.e., no CRS grade 2+). Such a clean ‘CRS negative cohort’ requires separating all ambiguous cases or those with missing data. Fever has a central role in CRS grading. Hence, we first excluded patients with missing evidence of fever. For the ’strict’ and ‘extended’ definitions, patients without temperature measurement were excluded, while for the ‘extended+mitigation’ definition, patients without temperature measurement were still included if they received fever mitigation. Next, we separated grade 0 and grade 1 patients as ‘definite grade 0 or 1’ if measurements/data on hypotension or hypoxia was available, but consistently in the normal range, or ‘probable grade 0 or 1’ if hypotension or hypoxia measurements were unavailable (assuming that they would have been reported if the patient had been severely ill). The ‘probable’ or ‘definite’ separation enables more granularity or to exclude probable graded patients in further analysis. Finally, patients with partial features of grade 2+, but who did not meet the grade 1 criterion (e.g., cardiovascular instability in the absence of fever) were labeled as ‘ambiguous’ and consequently excluded from grading since the guideline does not consider those cases and there is no evidence of inflammatory etiology for the symptoms (see discussion).

Altogether, we propose a general EHR ASTCT grading strategy that accounts for the “grade N+” classification of patients, and additionally enables the definition of ‘CRS positive’ and ‘CRS negative’ cohorts from EHR datasets.

### CRS grading algorithm identifies COVID-19 and TCE patients with different CRS grades

We applied the decision tree illustrated in Figure 2B to the three cohorts of interest (COVID-19 adult, TCE adult and TCE pediatric).

**Figure 3:**
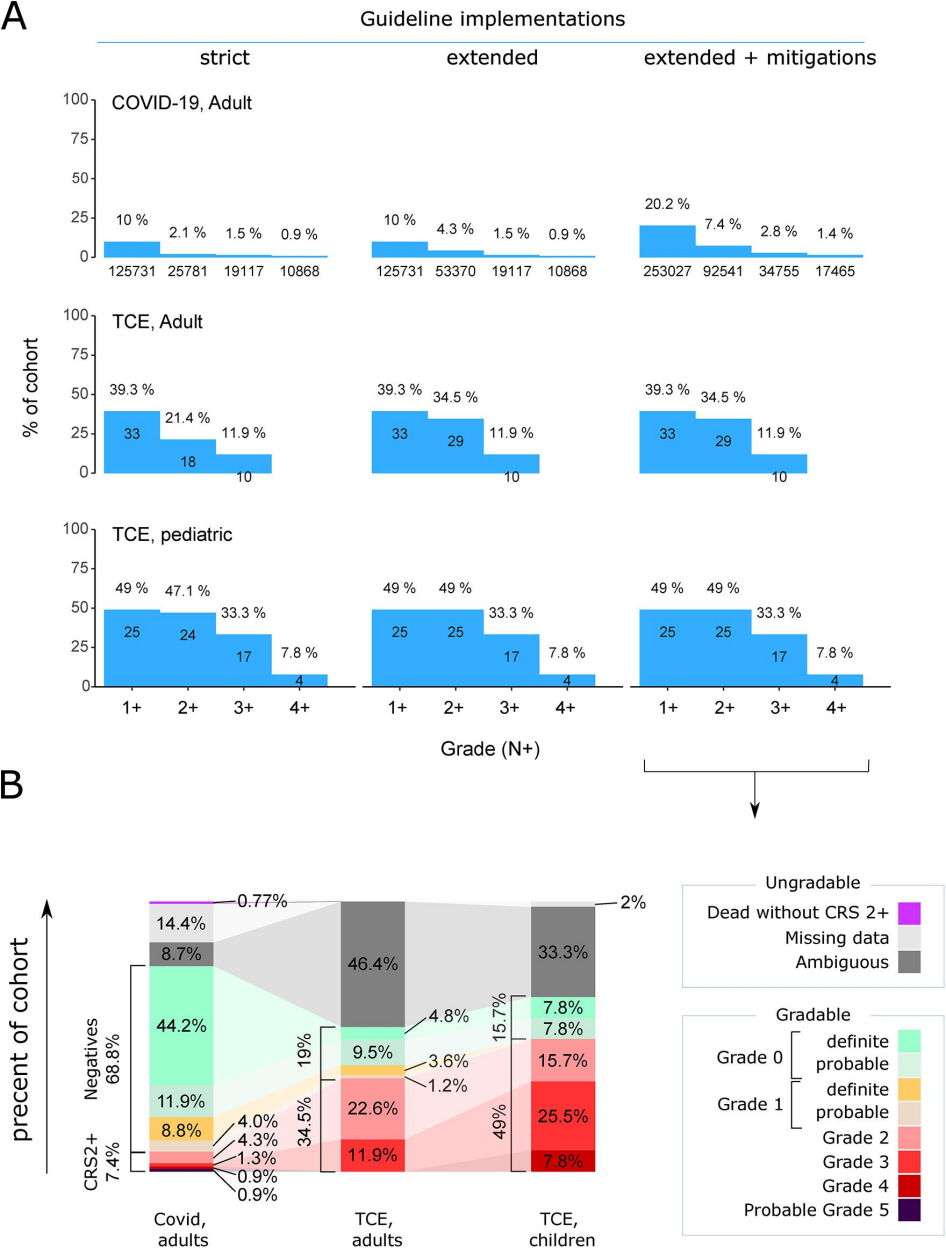
Identified patients following different implementations of the consensus ASTCT CRS grading on the COVID-19 and TCE cohorts. **(A)** Grading into N + groups, from grade 1+ to grade 4+, depending on the implementations: strict, extended, extended+mitigations. **(B)** Breakdown of the cohorts by grades based on the ‘extended+mitigations’ implementation, including details of definite and probable grading, as well as ambiguous patients who show symptoms of a higher grade but do not qualify for grade 1, and deceased patients with (probable grade 5) or without (not gradable) grade 2+ features. Notably, sepsis patients have been excluded, and the cohort includes patients who could be CRS positive or negative. Therefore, the percentages are calculated within the “usable cohort” of patients who did not experience sepsis, but these numbers should be adjusted to include the entire cohort, including sepsis cases, if prevalence needs to be determined.

Using the strict implementation, we identified 125,731, 25,781, 19,117, and 10,868 COVID-19 patients with grade 1+ to 4+, respectively (Figure 3A, left column). Among the 26,068 patients who died within one month of their COVID-19 diagnosis, 6,025 (23.1%) qualified for grade 2+, and 5,037 also qualified for grade 3+ (19.3%). Notably, many more patients were monitored for hypoxia using pulse oximetry SpO2 (361,429 monitored COVID-19 patients within 30 days) compared to arterial oxygen measurement SaO2 (33,299 monitored COVID-19 patients). SaO2 is measured invasively and usually indicates a worse patient condition in the intensive care unit, especially when they are on or near ventilation requirements. By allowing the use of SpO2 along with PvO2 and PaO2 in addition to SaO2 (‘extended definition’) to detect hypoxia, we identified 125,731, 53,370, 19,117, and 10,868 patients with grade 1+ to 4+, respectively. (Figure 3A, middle column). Finally, we applied the “extended+mitigations” implementation. This stratgy identified 253,027, 92,541, 34,755 and 17,465 patients with grades 1+ to 4+, respectively (Figure 3A, right column). Therefore, the sensitivity of CRS case identification can be increased by expanding the list of features used.

In the adult TCE cohort, all adult patients had their temperature measured. Following the strict CRS definition (Figure 3A, left column), 33 had only fever detected (grade 1+), 18 patients were assigned grade 2+, and 10 grade 3+ CRS. Using the extended definition (Figure 3A, middle column), 29 cases of grade 2+ and 10 of grade 3+ were identified. The grading was unchanged when fever mitigation was included as a criterion (Figure 3A, right column), indicating that those receiving fever mitigation reportedly also had fever.

TCE pediatric patients showed a similar distribution of CRS grading as adult patients: 25 had grade 1+, 24 patients had grade 2+ CRS, and 17 patients had grade 3+ CRS. Four patients were labeled with grade 4+ CRS across all implementations. Only one patient had missing temperature measurements, but was detected using the extended CRS definition for a grade 2. As in the adult TCE cohort, all patients with fever mitigation also had reported fever.

In TCE, the number of patients with each grade was similar across definitions, while there were higher discrepancies between definitions in the COVID-19 cohort. This can be attributed to the higher or more consistent monitoring in TCE patients, as well as the fact that the grading system has been developed to best describe immunotherapy-induced CRS. Moreover, COVID-19 patients may partially fulfill grade 1–2 criteria without having true CRS, for instance, hypoxemia due to pneumonic infiltrations and not associated with systemic capillary leakage (see discussion).

The breakdown of patients into each grade as well as into CRS positive and negative cohorts is shown in Figure 3B (Supplementary Table S1 for details) using the “extended+mitigations” definition.

In TCE adult patients, the grade 2+ positive cohort contained 29 patients (34.5%), while the negative cohort contained 16 (19%) patients, after exclusion of 39 (46.4%) ambiguously classified patients. A grade 3+ based cohort contained 10 (11.9%) positives and 35 (41.7%) negatives. For TCE pediatric patients, the grade 2+ based cohort contained 25 (49%) positives and 8 (15.7%) negatives, compared to 17 (33.3%) positives and 16 (31.3%) negatives for a grade 3+ based cohort. One patient with missing data and 17 (33.3%) ambiguous patients were removed.

In COVID-19 patients, we identified a cohort of 92 541 patients fulfilling the CRS grade 2+ definition (7.4%) and 862 707 patients (68.8%) that comply with grade 0 or 1. A total of 180 097 (14.4%) patients data needed to be discarded because of missing data on fever or mitigation [180 097 (14.4%)], ambiguous grading 108 585 (8.7%), or deceased patients without evidence for grade 2+ features before death 9,702 (0.77%). Using grade 3+ as the cut-off for a more severely ill cohort, we identified 34 755 (2.7%) COVID-19 patients and complemented by 917 235 (73.2%) who did not fulfill the CRS 3+ definition.

The high amount of ambiguous patients in each cohort highlights the need to define negatives and exact grades before building positive and negative cohorts. The fact that we could identify a substantial number of negative patients shows that comparing CRS positive and negative patients is feasible using EHR datasets.

### CRS grading only partially overlaps with ICD labels—discordance identified between the two gradings

We next compared the CRS grading according to our case identification algorithm vs. the available ICD codes that include a grading (Supplementary Table S1, Supplementary File 1).

Among the 1,100 COVID-19 patients with an ICD code for CRS without a specified grade, 795 (72.2%) were assigned to grade 2+ CRS; 105 (9.5%) to grade 1+, and 34 (3%) to grade 0, based on the algorithm. This validates our CRS grading implementation for grades 2 and higher in COVID-19 patients. Among the 56 patients with an ICD code for grade 2+, 33 (58.9%) were assigned to grade 2+, while 18 (32%) were assigned a grade of 0 or 1 based on the data in the EHR. Conversely, out of 92,541 patients assigned grade 2+ based on our analysis, only 835 had a CRS ICD label (with or without grade), supporting that either clinicians did not label COVID-19 patients with CRS codes or that the ASTCT CRS over-identifies subjects as having CRS in the context of an infectious disease, such as COVID-19.

In the TCE cohort, the number of patients with ICD-labeled grade was too small to allow for meaningful statistical comparisons. Our guideline-based grading identified 29 and 25 grade 2+ patients in TCE adults and pediatric cases, respectively, which was more than double the number of cases with a respective ICD code for CRS (11 and 12 patients). In contrast, many patients showing evidence of CRS using the grading decision tree were not labeled with the corresponding ICD code in the EHR. Therefore, we find evidence that relying solely on CRS ICD codes may cause us to miss a significant number of cases.

### CRS grading does not overlap with inflammatory CRS definitions

Systemic inflammation is a hallmark of CRS. The ASTCT-based grading guideline, however, only considers fever as a definite sign of inflammation. Since other, non-inflammatory medical conditions may fulfill ASTCT CRS criteria, we next hypothesized that data-based CRS definitions that consider inflammatory biomarkers may also be relevant, especially for COVID-19. Particularly, we implemented three published CRS definitions for COVID-19 that are strongly relying on inflammatory markers: Cov-Hi (20), c-His (19), and the newest definition from Declercq (22). The time window of 30 days after COVID-19 diagnosis or TCE injection was considered, similar to the grading time window.

Using the ‘extended+mitigation’ implementation, from the 92,541 COVID-19 patients with grade 2+ (Supplementary Table S3), 49,078 (53.0%) had sufficient lab data available to be assessed for Declercq's inflammatory CRS definition. Only 17,019 (34.7%) of those subjects classified as grade 2+ according to the ASTCT definition also fulfilled the inflammatory definition. Within grade 4 CRS patients, only 43.3% were positive for Declercq's inflammatory CRS definition (Supplementary File 1). The other two inflammatory CRS definitions -Cov-Hi and c-His- showed the same pattern: 33.9% of ASTCT grade 2+ patients fulfilled the cov-Hi CRS criteria, and 43.1% the c-HIS criteria. (Supplementary File 1).

In the TCE adult cohort, there were always more patients classified as ‘inflammatory CRS definition’-negative than positive within the grade 2+ patients, independent of which of of the three inflammatory CRS definitions was used (Supplementary File 1). Based on the available data, only the c-HIS was applicable to TCE adult patients, where 18 of 25 grade 2+ patients (72.0%) did not fulfill the c-HIS criteria. Therefore, the majority of ASTCT CRS grade 2+ COVID-19 or TCE patients had normal inflammatory markers according to the tested definitions and available measurements.

### ASTCT CRS grades do not consistently correlate with markers of systemic inflammation

**Figure 4:**
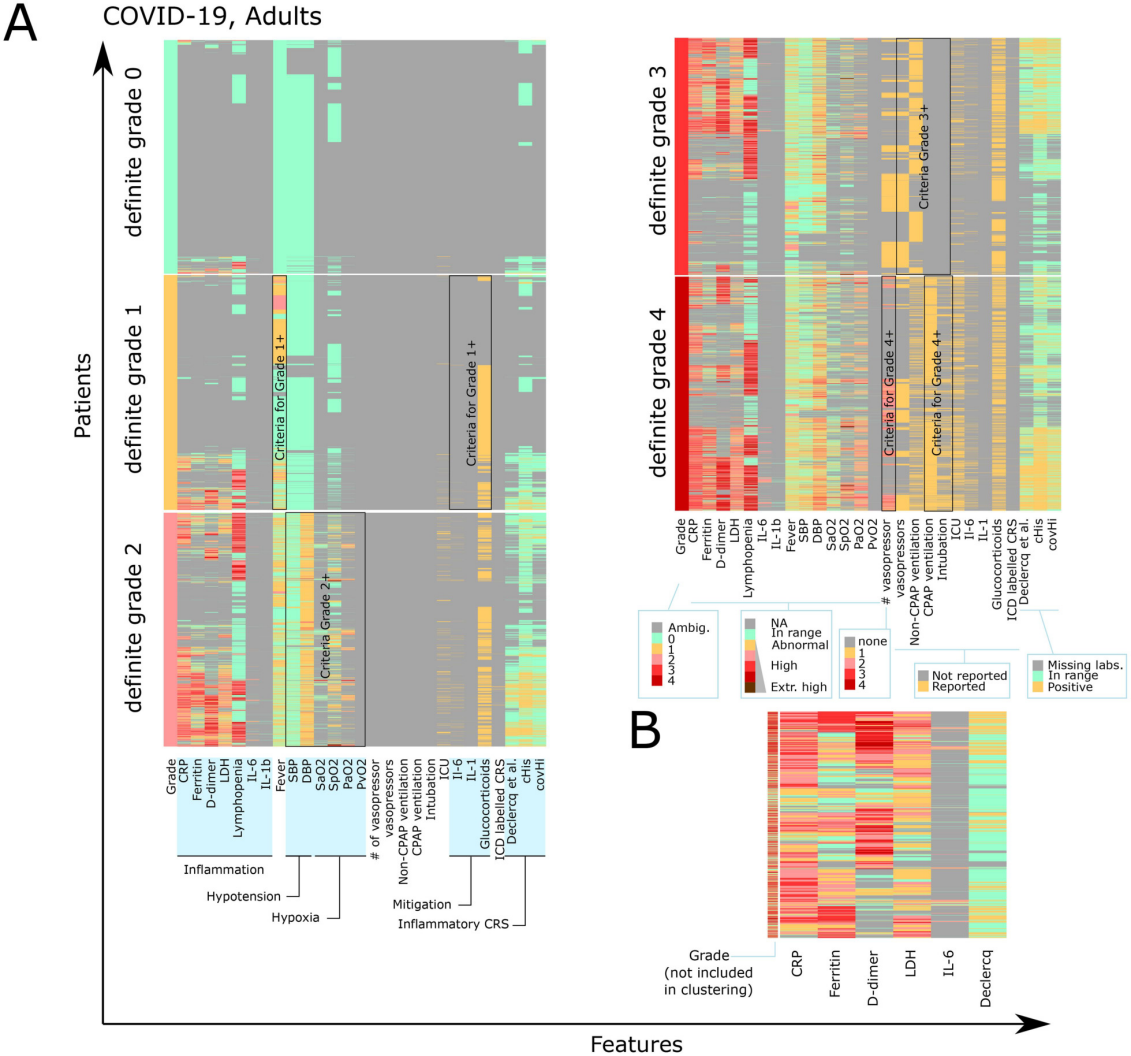
Overlap between patients of different grades and inflammatory markers. **(A)** Hierarchical clustering of patient features is performed separately within each grade, with one patient per row and one feature per column. Quantitative features are transformed into integer levels based on thresholds: not measured or not reported (−1, gray), normal range (green), and extreme high levels (dark red). See color scales for each group of features. The features used in the decision tree are shown as black boxes. CPAP ventilation can be invasive (intubation) or non-invasive. ICU: admitted to the intensive care unit. **(B)** Hierarchical clustering of patients was performed using only CRP, ferritin, D-dimer, LDH, IL-6, and Declercq's inflammatory CRS, with their grade labeled on the left. Only 5,000 individuals per definitive grade were included to ensure a balanced number of patients in each grade.

Next, we were interested in understanding whether systemic inflammation or the patterns of inflammatory markers in COVID-19 relate to the assigned ASTCT CRS grade. Based on data availability, we focused this analysis on COVID-19 patients. We extracted the peak value of commonly measured inflammation-related markers in COVID-19 patients: C-reactive protein (CRP), ferritin, Lactate Dehydrogenase (LDH), and D-dimers, and the lowest detected lymphocyte count (nadir of lymphocytopenia). These data were contrasted to the patient features used for the grading (i.e., the lowest measured systolic blood pressure or oxygen level), as well as the requirement for vasopressors, invasive or non-invasive ventilation, and intensive care. Patient profiles were clustered within each grade separately (Figure 4A). In patients in grades 0 and 1, very few had inflammatory markers that were out of range. Among patients with grade 2 to 4, CRP was generally high in most cases, while we consistently identified groups of patients with both high and low inflammatory profiles for other biomarkers, including positive and negative inflammatory CRS criteria. Cytokines such as IL-1 and IL-6 were rarely measured.

**Figure 5:**
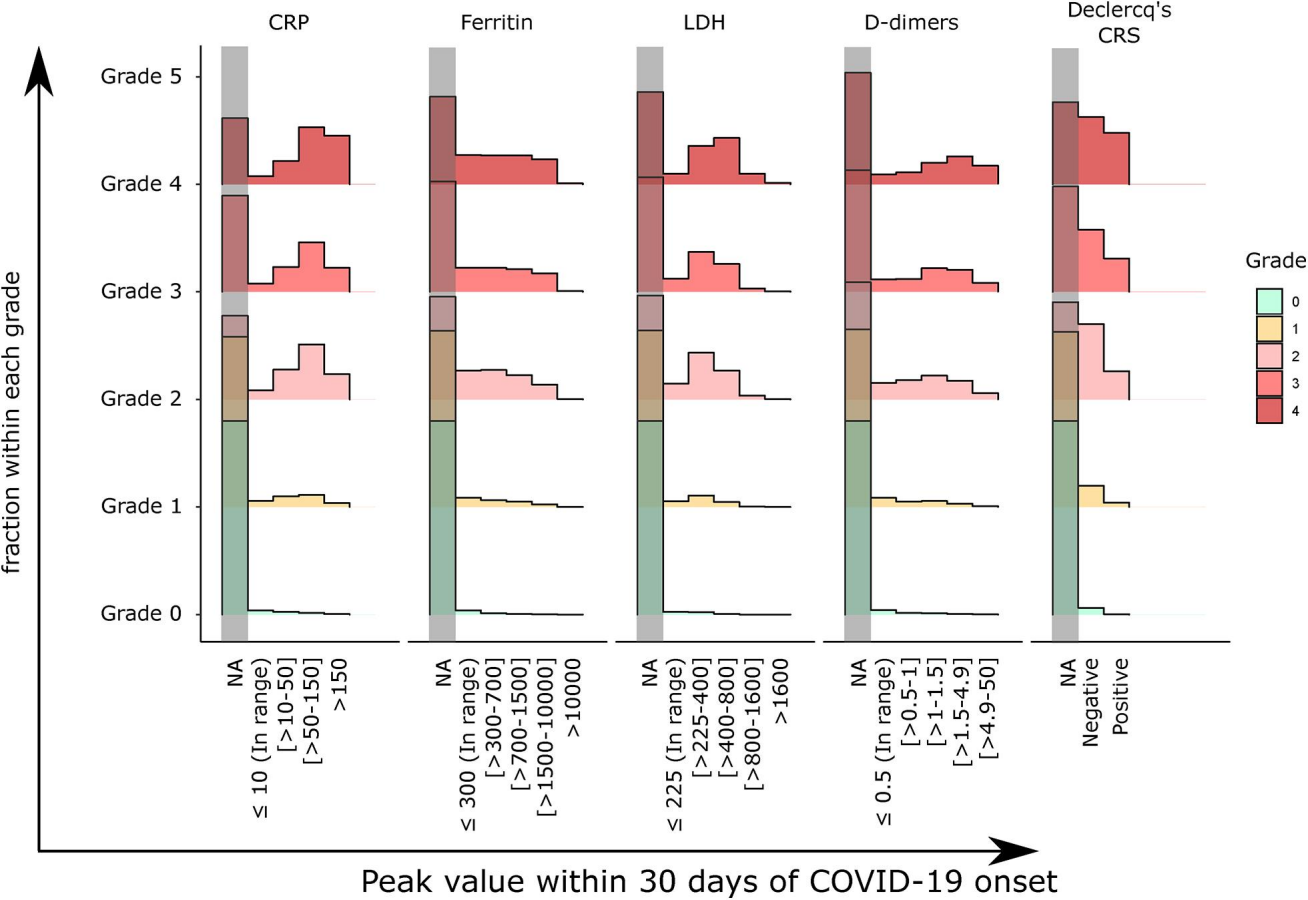
Distribution of single inflammatory markers within patients of each definite grade, separated into not measured (NA), in range, and different intensity groups. The groups match the color code used in panel A and B.

We confirmed this heterogeneity in a second analysis, where we randomly selected 5,000 patients within each definite grade and with at least one inflammatory measurement 30 days post-COVID-19 onset (CRP, LDH, ferritin, d-dimers, IL-6 or IL-1b) and clustered patients by these inflammatory markers (Figure 4B), without using the grade as information during clustering. We observed many combinations of inflammatory biomarkers, such as high D-dimers with high or low ferritin, and high or low LDH, and with high or low CRP. The subset of patients with consistently very high inflammatory values did not cluster by their grade. Additionally, the distribution of single inflammatory markers was similar between grades 2 and 4 (Figure 5). Using hierarchical clustering of individual longitudinal inflammatory markers, we confirmed that there is heterogeneity in patient dynamics for selected patient features, such as patients with shorter or more prolonged use of vasopressors (Supplementary Figure S1). Therefore, in infection, high inflammation does occur in some patients without features of CRS, and the direct application of CRS grading reflects the severity of disease but may not fully allow assigning whether hyperinflammation caused the severe condition, in which case a case identification strategy combining the proposed CRS grading and one inflammatory criteria such as the one from Declercq’ could be used instead (22).

The identification of cases that fulfill high-grade CRS but have inflammatory markers within the range supports the existence of patients with high and low inflammation within the same CRS grade. Strikingly, ‘hyperinflamed’ patients were not a homogenous group but showed different combinations of inflammatory marker patterns with either predominantly high CRP, ferritin, or LDH profiles, which could represent a sub-cohort of patients to be treated differently.

## Discussion

We evaluated the feasibility of identifying CRS patients within EHR datasets. EHR datasets offer a tremendous potential to detect specific profiles and trajectories of patients at risk for adverse events or positive treatment responses, in real-world settings, and with large datasets suitable for data-driven explorative approaches. However, the main challenge is achieving reliable case identification of patient patterns [“computable phenotypes” (27, 28)] and obtaining results that are robust to noise inherent in EHR, such as missing measurements or data. Additionally, there is widespread variation in how events are defined in EHR datasets, which makes studies difficult to compare or reproduce (29). These challenges are even more pronounced in conditions like CRS, which is characterized by a complex mix of findings and symptoms that are not specific to CRS and depend on a series of causally related events.

We used the Optum® COVID-19 dataset to explore the feasibility and limitations of different case identification strategies and to compare the differences in labelled CRS patients between infectious and immunotherapy-induced syndromes. We observed that patient labeling with ICD codes for CRS generally did not consider the CRS grade, and many patients showed clinical and biomarker evidence of CRS without being ICD labeled as CRS. This highlights the need for evidence-based, objective criteria to define CRS and CRS grades in EHR. Others have recently explored a model to identify CRS in EHR based on grade-specific ICD codes and using a regression model to determine clinical features indicating CRS (30, 31). The authors suggested that hypotension, positive pressure ventilation, and the administration of tocilizumab or vasopressors could serve as signals of severe CRS in retrospective datasets. Notably, all these features were included in our algorithm. Various CRS criteria have been proposed, typically developed for specific conditions such as CAR-T treatment or COVID-19 infection, but it remains uncertain if these definitions can be used interchangeably.

We validated this by proposing a practical implementation of the CRS grading consensus guideline (17) that can handle some levels of missing data as well as inflammatory CRS criteria. Specifically, we used different implementation strategies for the grades with varying levels of complexity and suggested a setting where we demonstrated capturing most cases with CRS codes in the EHR using our algorithm. We then extracted both patients with positive evidence for each CRS grade and respective negative cohorts without hypotension, hypoxia, and/or fever.

### Limitations

Missing data is an intrinsic limitation of EHR datasets. We have minimized the risk of inaccurate CRS grade assignment due to missing data by establishing a gray zone for patients with ambiguous data. These patients either lack temperature measurements or have ambiguous findings that could suggest a higher grade CRS but do not meet the criteria for grade 2+, leading to uncertainties that resulted in their exclusion from further analysis. Since ambiguous patients showed internally inconsistent findings (e.g., isolated hypotension without fever or inflammatory markers), their incomplete data prevented meaningful comparison with graded patients and did not meet the diagnostic criteria for CRS. We did not observe a specific bias toward a particular age or gender distribution in these patients (Supplementary Figure S2) and excluded them from the gradings. By removing these gray zone patients, we reduce the statistical power of further cohort analyses, which was partly offset by the large number of COVID-19 patients, but it limited our analysis of TCE-treated patients in this dataset.

Further, our tunable grading decision tree allows us to test the sensitivity of grading decisions in downstream tasks such as prediction of CRS risk. Notably, we are not introducing a new CRS grading; however, we enable the application of the ASTCT grading guideline to EHR datasets in a flexible manner, allowing for both the consideration of ambiguities in the guideline and the handling of missing information in the datasets. When defining positive and negative cohorts, patients with ICD codes for grade 2+ (or grade 3+) can also be added to the positive class.

### Absence of ground-truth validation dataset

There was only a partial overlap between CRS 2+ patients and ICD-labeled patients. Since there is no accepted ground truth real-world dataset for CRS patients, it is challenging to dissect why some patients were ICD labeled as CRS (with or without a coded grade) but were classified as being grade 0 or grade 1 by the decision tree. Delays or inaccuracies in adopting the ICD code in non-oncology-related fields; missing reported data at the time of presumed CRS, or grading following older guidelines may have contributed to these inconsistencies. Therefore, assessing the performance of the case identification algorithm using ICD codes for CRS grades as a potential ground-truth reference is not possible since the data we used as evidence for grading and the CRS ICD codes themselves have the same shortcoming of potential missing patient labels. Instead, one would need an EHR dataset combined with a clinically validated annotation of CRS grades, for instance, a chart review using a clinician-assisted search of clinical notes related to the same patients present in the EHR dataset. Such a dataset would provide a ground-truth benchmark to compare the performance of different case identification strategies in the decision tree. An important next step will be to apply the algorithm across additional EHR datasets to further explore generalizability. Currently, we focused on developing and stress-testing the algorithm within a large, well-characterized cohort because other datasets lack clinician-validated CRS labels that allow full validation of the case identification algorithm, differ substantially in coding structures, and involve significant acquisition costs.

### Comparison of CRS prevalence and impact of definitions

In the TCE cohorts, the different grading implementations raised similar results and we observed a relatively high prevalence of patients with grade 2+ CRS in the TCE cohort: 29 of 84 adults (34%) after excluding sepsis, i.e., 29 of 106 adult patients (27.3%) compared to expected clinical results [11%–15% (23)]. First, we do not know the origin of the TCE patients and we cannot exclude that this specific TCE cohort within the Optum COVID-19 data may derive from a study focusing on patients with specific comorbidities. Second, the definition of grade 2+ has ambiguities in the guideline. It might be that patients that we identify grade 2+ would not be labeled grade 2+ by a clinician if the hypotension/hypoxia is too short, or if there might be another reason for the hypotension/hypoxia. A sensitivity analysis (Figure 3A) demonstrating the high dependence of case prevalence on the specific clinical features (e.g., SaO2 vs. SpO2) and guideline interpretations (e.g., inclusion of fever mitigation) employed: Definitions using more lab values (‘extended’) and using fever mitigation identified many more patients than only using the minimal (‘strict’) set of evidence required. This could be due to ICD diagnoses not reliably entered, lab values not being consistently measured, or that the grading definitions become too broad in the context of COVID-19 infection, as high-graded patients could represent a multimorbid patient subset without hyperinflammation. Overall, the small size of our TCE cohort limits a comprehensive epidemiological assessment of the high CRS incidence observed in blinatumomab patients, but it demonstrates the algorithm's applicability to different conditions that can cause CRS in future datasets.

### Orthogonality between inflammatory levels and CRS grading

We found that CRS grade according to ASTCT guidelines and inflammation were dissociated in COVID-19 patients. Patients with a high- and low- inflammatory phenotype were represented in the groups that were graded with grade 3 or 4 according to the CRS definition. As previously suggested, the low inflammation phenotype that is compliant with a high CRS grade might indicate cases of severe COVID-19 with respiratory or cardiovascular failure not due to cytokine release, or those that were strongly mitigated. However, and despite the small cohort size, we observed the same phenomenon in TCE patients with poor overlap between grading and inflammatory criteria, suggesting that some patients have true CRS with low inflammation with potential clinical consequences, as they may be more or less responsive to cytokine blockers or other anti-inflammatory treatments.

Recent clinical and mechanistic studies established that severe COVID-19 represents a heterogeneous clinical state driven not only by hyperinflammation but also by pre-existing organ dysfunction and infection-induced tissue injury. The hyperinflammatory response is rooted in early impairment of type I interferon signaling, which enables viral persistence and overactivation of macrophages and neutrophils (32, 33). This innate activation—amplified by NLRP3 inflammasome (34) signaling, pyroptosis, NET formation, endothelial dysfunction, and complement dysregulation—produces high levels of proinflammatory cytokines and promotes microvascular injury and coagulopathy. Within this milieu, hyperinflammation is further shaped by T-cell lymphopenia, Th17-skewing (Li et al., Sci Bull), senescent effector CD8^+^ T cells, and dendritic-cell dysfunction, all of which perpetuate immune imbalance. At the molecular level, disease severity is associated with dysregulated alternative splicing, altered autophagy programs linked to ESCRT-complex activity in monocytes (35), and shifts in m⁶A RNA-modification regulators that track closely with clinical outcomes (36).

The high diversity of inflammatory profiles suggests that CRS may be driven by distinct pathways, monitored downstream here by high ferritin, or high d-dimers, or LDH, and may suggest to use patient-tailored mitigation strategies to the relevant active pathway. CRP and ferritin are acute phase reactants that are synthesized in response to proinflammatory cytokine release. Thus, they are surrogate markers for a previous IL-6 increase. Hyperferritinemia reflects macrophage-activation-driven inflammation, in which TNF and IFN-*γ* are critical cytokines. LDH increases are typically observed following cytotoxicity and cytolysis, which can also occur in patients undergoing immunotherapy treatment due to tumor lysis. Thus, high LDH levels reflect high tissue damage, which can be either hypoxic due to low blood pressure or due to cytotoxicity of CD8 T-cells. The current analysis does not capture the full dynamics of such CRS inflammatory markers, and might hide inflammatory processes with different kinetics measured with poor time resolution. The existence of different inflammatory profiles suggests that these profiles may potentially guide personalized treatment choices to suppress hyperinflammation in severe COVID-19 or other CRS. This should be further explored in prospective studies with more dense time reporting and predefined treatment strategies.

CRS case identification will be essential for comparing the trajectories of patients with CRS from different conditions and assessing which features and risk factors are transferable between them. For instance, COVID-19 patients show different cytokine levels than CAR-T and sepsis (4, 37, 38) or Hemophagocytic lymphohistiocytosis (HLH) (39). Previous scores on other Cytokine Storm Syndromes (CSS) do not directly align with inflammatory CRS definitions (40, 41). Further, single inflammatory markers have been poorly predictive of CRS severity (42), which is consistent with our observation that half of the patients with severe CRS grades show some inflammatory markers in the normal range. Altogether, our results suggest multiple independent components of CRS trajectories, where grading would only constitute one dimension, complemented by inflammatory markers and potentially survival. Either defining subgrades with or without inflammation, or predicting separately risk factors for high inflammation or severe grade would allow disentangling those components of CRS.

### Possibility to refine the gradings

Here, we stayed within the application of already published and accepted definitions of CRS, either grading-based or inflammation-based. The discrepancies between the two definitions suggest that, for the future, EHR databases can be used to combine CRS features in a more precise manner or to define more stratified sub-patient profiles, rather than relying solely on grades or inflammatory status. First, one could include ICD-coded CRS patients in the positive CRS cohort despite the lack of reported evidence. Second, COVID-19 patients could be separated into inflammatory severe CRS (grade 2+ or 3+) by requiring high inflammation to be qualified for ‘real’ CRS instead of, e.g., severe lung damage or heart failure. Third, the diversity of available codes in EHR datasets opens the opportunity to refine sub-stratifications of the grades, either by including information on the type of ventilation (CPAP or intubation), the severity of symptoms (such as level of hypoxia or additional neurotoxicity) and the strength of mitigations (such as the dose of vasopressors or the cytokine blocking strategy used). Comparing grading with lab values measured in the first days after the triggering event or after fever onset may determine which lab values are predictive for CRS (early response prediction), which would require a more detailed dynamical analysis between grading dynamics and inflammatory dynamics. The combination of mitigation and lab values might reveal different classes of severity by differential response to mitigation. We have also identified a gray zone of ambiguous patients with severe symptoms without grade 1 in both COVID-19 and TCE patients, which highlights the need for careful negative cohort design. It is not clear if those patients lack reported data for temperature, if other fever mitigations strategies (not included in our grading such as ibuprofen or paracetamol) were efficient in those patients to mask fever, or if these patients developed CRS without fever, which was sometimes reported for CRS (43), and in the context of systemic inflammation where some patients become hypothermic (44). Therefore, these ambiguous patients may reveal a gray zone where the guideline cannot yet be applied, and could suggest a refinement of the guideline.

In the context of TCE, our study suggests that, due to the presence of ambiguous patients, large cohorts will be needed to extract CRS features with statistical features in EHR datasets. Further, the small number of negative patients reveals that despite severe CRS, many patients experience partial symptoms that would not qualify for negatives nor for positive, and this gray zone of patients may have influence on CRS insights gained in cohort comparison depending on if a study includes or excludes them from further analyses. It is important to know in advance such limitations of EHR datasets.

### General applicability

We proposed an implementation of the grading guideline (‘extended+mitigations’) according to standard clinical thresholds, and only considered published CRS definitions. This data pipeline and strategy is extendable to test other datasets or definitions of CRS. Although a larger range of fever mitigations could be considered, we focused on glucocorticoids and cytokine blockers as they have a substantial effect on fever and the choice of administering such strong fever mitigation suggests the patient had inflammatory symptoms. Our CRS identification strategy is directly extendable to different lab value thresholds that may vary in the context of pediatric, oncological, intensive care, and respiratory practices. Our decision tree can also be refined to define hypotension and hypoxia compared to the baseline tension or oxygen levels of patients before onset, to account for pre-existing comorbidities, and to consider temporal features such as a minimum duration of hypoxia, hypotension, vasopressor or ventilation usage.

### General implications for mitigation strategies

Although the direct identification of which patient received separate mitigation strategies and how they responded to treatments is outside the current scope, we have demonstrated a proof of concept that EHR datasets can be leveraged to provide insights into possible patient stratification, thanks to the large amount of time-course data. Analyses of CRS outcomes in patients who received anti-inflammatory treatments might help determine if inflammatory stratification correlates with response to mitigation. However, since more severely ill patients are more likely to be given anti-inflammatory treatments, careful matching of patients at the same CRS stage and inflammation level before treatment is necessary. This requires an extended dynamic analysis that is outside the scope of the present study.

Altogether, we showed a proof of concept that CRS patients can be identified in EHR datasets such as the Optum® COVID-19 data. We provide a strategy for performing a grading with well-defined positives and negatives, which is critical to overcome missing data and noise in EHR datasets and get high quality analyses. Of note, this approach is directly translatable to the commonly-used EHR databases, such as TrinetX or IBM Marketscan.

## Methods

### Optum® COVID-19 data and general cleaning pipeline

The Optum® COVID-19 data cross-references patient trajectories recorded through health insurance claims, pharmacy claims data, lab test results, inpatient confinement data, and provider data. Roche acquired the license to access this dataset until April 15, 2023. The dataset contains 16 tables describing the health history of 12,306,101 patients across the USA. We focused our analyses on the tables: “diagnostics”, “procedures”, “med_administrations”, “patient_reported_med”, “prescriptions”, “labs”, which contains lab results, and “Patients” that provide information on birth date, gender and if applicable, the month of death.

### Definition of COVID-19 positive subjects and time of onset

For the COVID-19 cohort, we isolated only subjects with active COVID-19 infection. During the early months of the pandemic, with lower testing available, COVID-19 infection was defined using diagnosis codes reporting pneumonia or emergency situation due to COVID-19 infection: B342 (unspecified coronavirus infection), B972, B9721, B9729 (coronavirus as the cause of disease), J1281 or J1282 (pneumonia due to coronavirus). Later, since qPCR or antigen test results were directly reported as lab values, we additionally selected patients with a “positive” result for one of the following test codes: “SARS coronavirus 2 RNA (COVID-19).unspecified specimen” (code 94309-2), “SARS coronavirus 2 RNA (COVID-19).respiratory” (code 94500-6), “SARS coronavirus 2 antigen (COVID-19).respiratory”**, “**SARS coronavirus 2 RNA (COVID-19).oral”, “SARS coronavirus RNA”, and “sars-cov-2; naa”. The qPCR cycle threshold (Ct) values were inconsistently available, and including Ct values as an additional way to identify cases did not increase the cohort size. We therefore excluded the lab values for qPCR Ct values. Finally, codes reporting the presence of antibodies against COVID-19 were not considered because they do not inform on the time of infection. For each patient, the earliest COVID-19 code as listed above is defined as the “onset time” of COVID-19 infection.

### Definition of TCE positive subjects and time of onset

We used ‘procedure’ and ‘medication’ codes to identify TCE patients. Blinatumomab was the only TCE treatment that could be identified, and it was obtained only from the medication tables using the NDC code 55513016001. No procedure code for blinatumomab nor medication codes for other TCE were found. We only considered the first TCE injection as a triggering event as the CRS risk is highest following the first TCE injection (45).

### Extraction of patient features and events around a time window of the triggering event

Since EHR reporting codes can be modified up to seven days after an event, and because we could not obtain definitive data on how long a patient was infected with SARS-CoV-2 before the code was entered, we considered patient features within a time window of 7 days before and up to 30 days after the coding in our analysis for CRS.

### Exclusion criteria

A critical step to define CRS in databases is to exclude medical conditions causing similar symptoms or findings. Sepsis is one of the prominent exclusion criterion for CRS. Patients receiving immunotherapy are at higher risk of sepsis due to immunodepletion, neutropenic fever, or overall poor immune function, and may experience non-CRS related sepsis after TCE treatment. Therefore, we exclude patients diagnosed with sepsis using ICD codes A40 (streptococcal sepsis), A41 (other sepsis), and all related downstream codes within the analysis window of [-7 days, +30 days].

Additionally, we exclude patients with prior experience (< 7 days before the triggering event) of code E83.11 (hemochromatosis) since these patients tend to have consistently high ferritin levels, which could confound inflammatory CRS criteria. We also exclude subjects with a prior diagnosis of code D76 (Hemophagocytic syndromes) and its subcodes, as well as M06.1 (Adult-onset Still's disease), which are autoinflammatory conditions that typically present as cytokine storm syndromes.

### General CRS identification pipeline

We created a mapping between every single criterion of the CRS grading scheme or inflammatory CRS definition (hypoxia, hypotension, etc.), to the medical reported codes and their description in the Optum® COVID-19 data (Supplementary File 2, Supplementary File 3). We separated feature groups (like ‘vasopressor’) from the more precise feature name (‘epinephrine’) from its coding (a list of NDC codes in the medication tables).

Finally, feature names were aggregated for their peak or nadir value over the [−7 days, 30 days] time-window of analysis and transformed into a binary value for the presence of each considered diagnosis, procedure or medication code, and a discrete integer scale for lab values, where −1 means non-measured, 0 means in range according to the University Hospital Basel range definitions (https://www.unispital-basel.ch/analysenverzeichnis), and higher values were determined based on logarithmic-scaled threshold (for instance those shown in Figure 5).

### Implementation of inflammatory CRS definitions

Multiple inflammatory CRS criteria have been defined, earlier in the context of CRS, such as HLH, and more recently in the context of COVID-19 infection. We implemented the latest CRS criteria (22) that combines inflammatory markers (CRP, Ferritin, LDH and D-dimer), lymphopenia and ventilation. This CRS definition requires either: 1/ a ferritin level higher than 2,000 ng/mL and ventilation of any type; 2/ an increasing level of ferritin within 24 h starting from at least 1,000 ng/mL; or 3/ lymphopenia (<800 cells/μL) in combination with either three inflammatory markers above threshold or two above threshold and increasing over 24 h. The considered markers and threshold were: Ferritin >700 ng/mL, D-dimer (FEU) > 1 mg/L, LDH >300 U/L, or CRP >70 mg/L.

As a comparison, the cov-HI criterion (20) was implemented as ferritin > 1,500 ng/mL or CRP > 150 mg/L or CRP >50 mg/L doubling within 24 h. The c-HIS criterion (19) was positive when two out of six criteria were present: 1/ fever > 38 °C, 2/ ferritin >700 ng/mL, 3/ CRP ≥ 150 mg/L or IL6 > 15 pg/mL or triglyceride ≥ 150 mg/dL, 4/ hemoglobin ≤ 9.2 g/dL or platelets ≤ 110,000/ul, 5/ d-dimer (FEU) ≥ 1.5 mg/L, 6/ LDH ≥ 400 U/L or AST ≥ 100 U/L.

## Data Availability

The R markdown scripts used for code extraction from an Optum SQL database up to the grading of individual patients and reproduction of the main plots of the article are available in an online repository (https://doi.org/10.5281/zenodo.17153356). Further inquiries can be directed to the corresponding author(s).
