# Supplementary material for: Guideline-based strategies to identify severe cytokine release syndrome in COVID-19 and cancer immunotherapy using large-scale electronic health records
Source: Front Digit Health. 2026 Feb 17;7:1625889. doi: 10.3389/fdgth.2025.1625889 (PMC12953395; doi:10.3389/fdgth.2025.1625889)
Supplement: Supplementary file 2 [file Table2.docx]

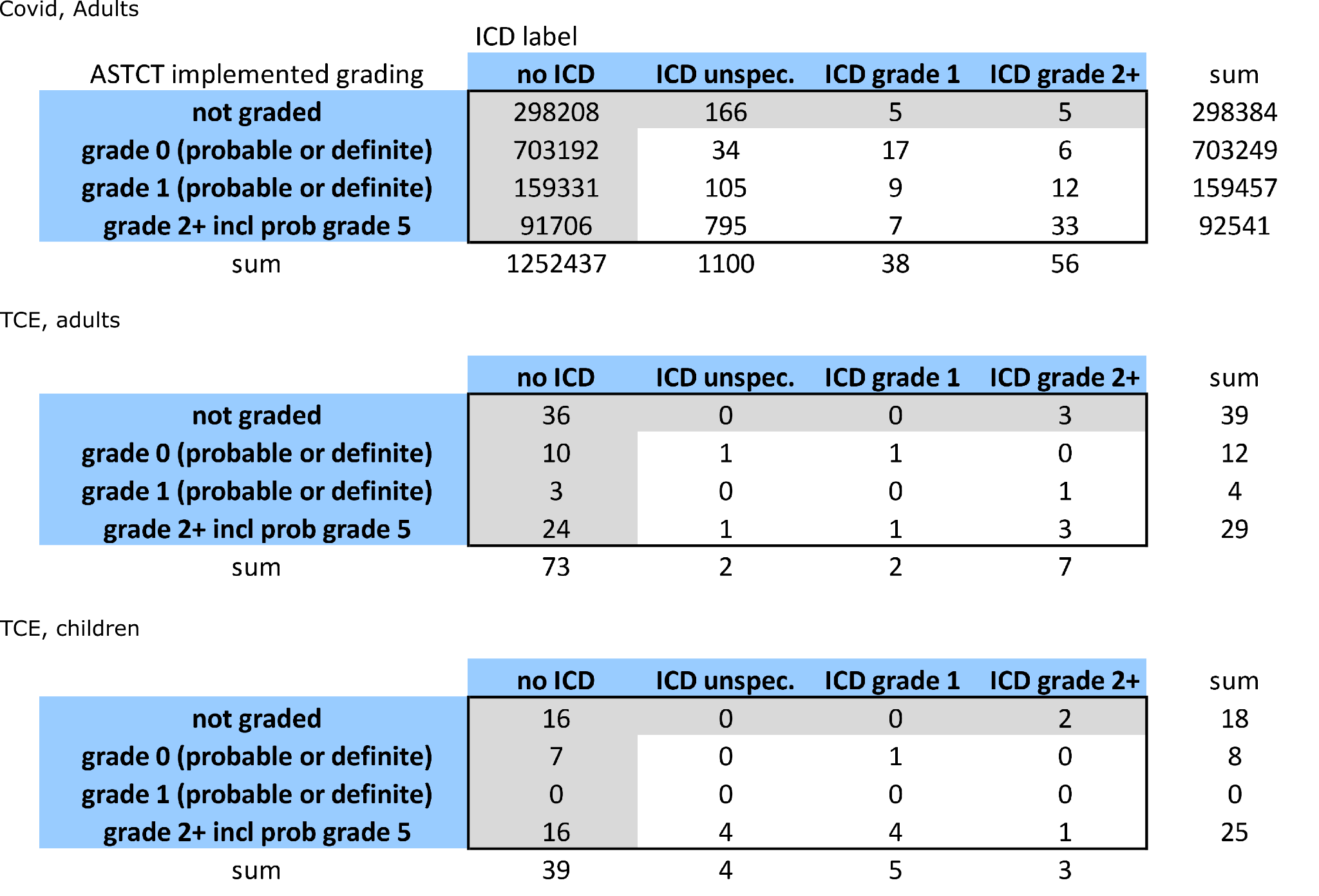


**Supplementary Table 2:** Overlap between the implemented ASTCT CRS grading and the use of ICD CRS label codes.
