# Supplementary material for: Guideline-based strategies to identify severe cytokine release syndrome in COVID-19 and cancer immunotherapy using large-scale electronic health records
Source: Front Digit Health. 2026 Feb 17;7:1625889. doi: 10.3389/fdgth.2025.1625889 (PMC12953395; doi:10.3389/fdgth.2025.1625889)
Supplement: Supplementary file 4 [file Table4.docx]

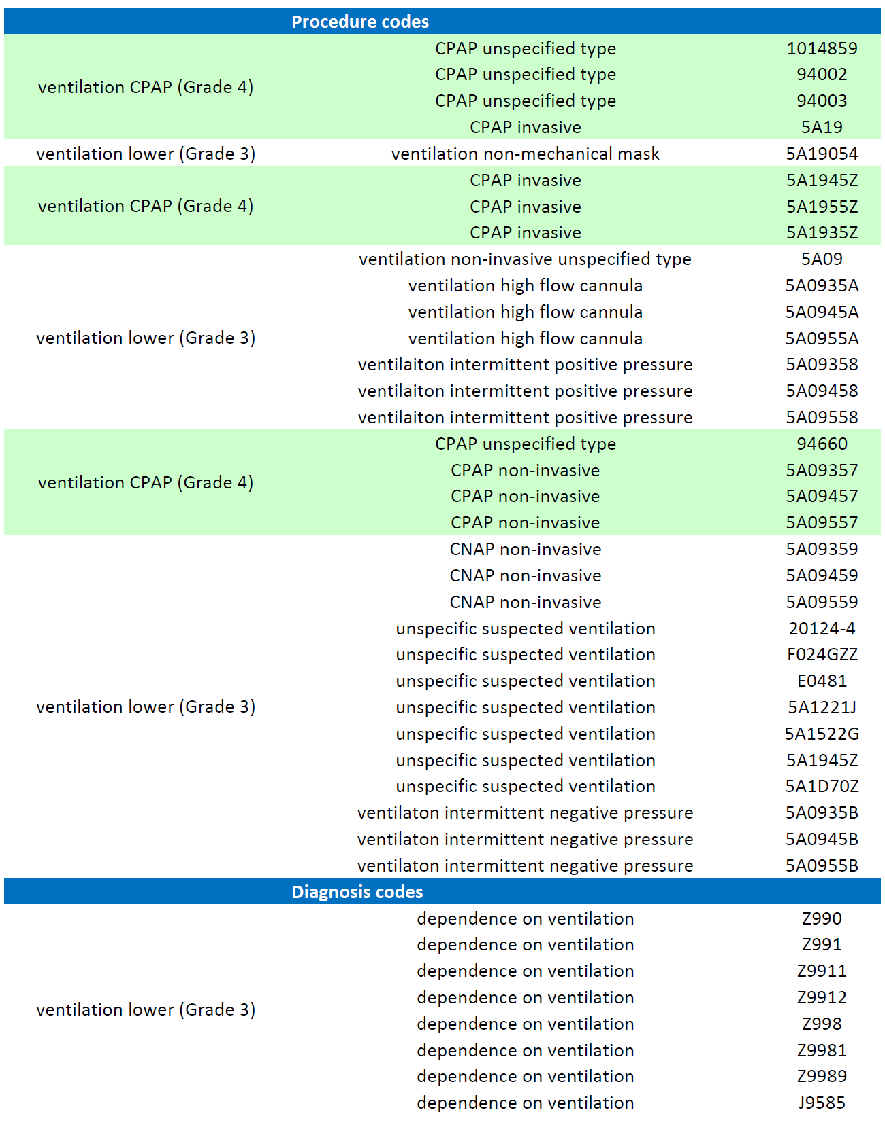

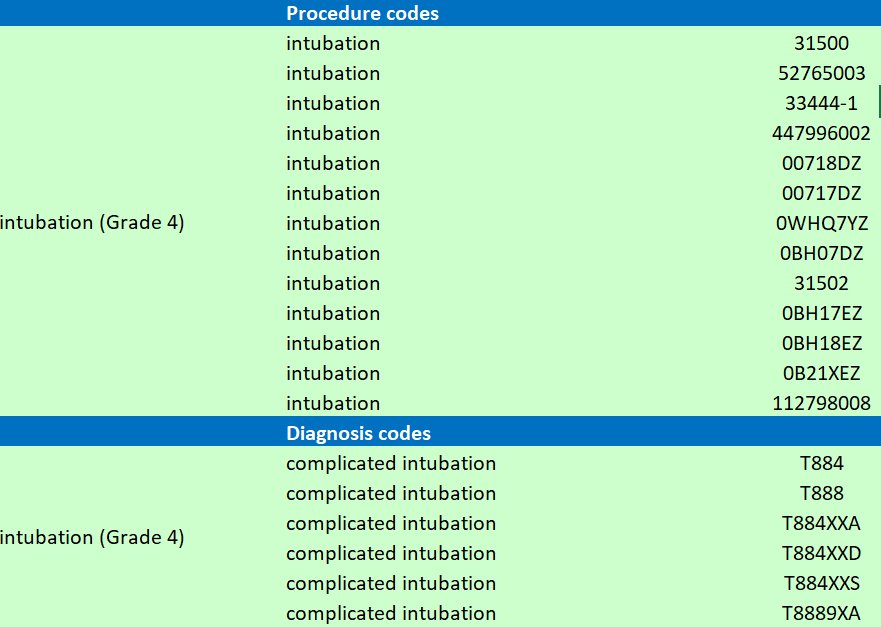


**Table S4: Link between procedure and diagnosis codes to the grading in the ASTCT consensus grading for detecting ventilation.** Green lines qualify for grade 4 (continuous pressure ventilation or higher: intubation). Evidence of other types of ventilation qualify for grade 3, as per the guideline.
