# Supplementary material for: Guideline-based strategies to identify severe cytokine release syndrome in COVID-19 and cancer immunotherapy using large-scale electronic health records
Source: Front Digit Health. 2026 Feb 17;7:1625889. doi: 10.3389/fdgth.2025.1625889 (PMC12953395; doi:10.3389/fdgth.2025.1625889)
Supplement: Supplementary file 6 [file Supplementaryfile2.pdf]

feature_group	feature_name	table	column	value	unit_column	unit_value	result_column
Inflammation	CRP	labs	test_name	C-reactive protein (CRP)	result_unit	mg/l	test_result
Inflammation	CRP	labs	test_name	C-reactive protein (CRP)	result_unit		test_result
Inflammation	HiCRP	labs	test_name	C-reactive protein.high-sensitivity (hsCRP)	result_unit	mg/l	test_result
Inflammation	HiCRP	labs	test_name	C-reactive protein.high-sensitivity (hsCRP)	result_unit		test_result
Inflammation	Ferritin	labs	test_name	Ferritin	result_unit	ng/ml	test_result
Inflammation	Ferritin	labs	test_name	Ferritin	result_unit		test_result
Liver	LDH	labs	test_name	Lactate dehydrogenase.total (LDH)	result_unit	u/l	test_result
Liver	LDH	labs	test_name	ldh	result_unit	u/l	test_result
Liver	LDH	labs	test_name	ldh bf	result_unit	u/l	test_result
BloodCells	Lymphocyte.absolute	labs	test_name	Lymphocyte.absolute	result_unit	x10^3/ul	test_result
BloodCells	Lymphocyte.absolute	labs	test_name	Lymphocyte.absolute	result_unit	thousands/l	test_result
BloodCells	Lymphocyte.absolute	labs	test_name	Lymphocyte.absolute	result_unit		test_result
Inflammation	Fibrinogen	labs	test_name	Fibrinogen	result_unit	mg/dl	test_result
Liver	ALT	labs	test_name	Alanine aminotransferase (ALT)	result_unit	u/l	test_result
Liver	AST	labs	test_name	Aspartate aminotransferase (AST)	result_unit	u/l	test_result
Liver	ALP	labs	test_name	Alkaline phosphatase.total (ALP)	result_unit	u/l	test_result
Inflammation	Creatine kinase.MB (CKMB).concentration	labs	test_name	Creatine kinase.MB (CKMB).concentration	result_unit	ng/ml	test_result
Inflammation	Creatine kinase.total (CK)	labs	test_name	Creatine kinase.total (CK)	result_unit	u/l	test_result
Inflammation	Creatinine	labs	test_name	Creatinine	result_unit	mg/dl	test_result
Inflammation	Creatinine clearance	labs	test_name	Creatinine clearance	result_unit	ml/min	test_result
Inflammation	Creatinine.urine.24 hour	labs	test_name	Creatinine.urine.24 hour	result_unit	g/24hr	test_result
Inflammation	Creatinine.urine.concentration	labs	test_name	Creatinine.urine.concentration	result_unit	mg/dl	test_result
Inflammation	Protein/creatinine ratio.urine	labs	test_name	Protein/creatinine ratio.urine	result_unit	mg/g	test_result
GeneralLabs	Blood urea nitrogen (BUN)	labs	test_name	Blood urea nitrogen (BUN)	result_unit	mg/dl	test_result
GeneralLabs	Blood urea nitrogen/creatinine ratio (BUN/CREA)	labs	test_name	Blood urea nitrogen/creatinine ratio (BUN/CREA)	result_unit	no units	test_result
GeneralLabs	Albumin	labs	test_name	Albumin	result_unit	g/dl	test_result
GeneralLabs	Albumin.urine.concentration	labs	test_name	Albumin.urine.concentration	result_unit	mg/dl	test_result
GeneralLabs	Albumin.urine.concentration	labs	test_name	Albumin.urine.concentration	result_unit		test_result
GeneralLabs	Albumin.urine.dipstick	labs	test_name	Albumin.urine.dipstick	result_unit	mg/l	test_result
GeneralLabs	Albumin.urine.dipstick	labs	test_name	Albumin.urine.dipstick	result_unit		test_result
GeneralLabs	Albumin/creatinine ratio.urine	labs	test_name	Albumin/creatinine ratio.urine	result_unit	mg/g	test_result
GeneralLabs	Albumin/creatinine ratio.urine	labs	test_name	Albumin/creatinine ratio.urine	result_unit	ug/mg	test_result
GeneralLabs	Albumin/Globulin	labs	test_name	Albumin/Globulin	result_unit		test_result
GeneralLabs	Albumin/Globulin	labs	test_name	Albumin/Globulin	result_unit	calc	test_result
GeneralLabs	Prealbumin (PALB)	labs	test_name	Prealbumin (PALB)	result_unit	mg/dl	test_result
GeneralLabs	Anion gap	labs	test_name	Anion gap	result_unit	mmol/l	test_result
GeneralLabs	Chloride (Cl)	labs	test_name	Chloride (Cl)	result_unit	mmol/l	test_result
GeneralLabs	Potassium (K)	labs	test_name	Potassium (K)	result_unit	mmol/l	test_result
GeneralLabs	Glucose.fasting	labs	test_name	Glucose.fasting	result_unit	mg/dl	test_result
GeneralLabs	Glucose.random	labs	test_name	Glucose.random	result_unit	mg/dl	test_result
GeneralLabs	Glucose.random	labs	test_name	Glucose.random	result_unit		test_result
GeneralLabs	Glucose.urine.dipstick	labs	test_name	Glucose.urine.dipstick	result_unit		test_result
GeneralLabs	Glucose.urine.dipstick	labs	test_name	Glucose.urine.dipstick	result_unit	mg/dl	test_result
Cytokines	Interleukin 1 beta (IL-1B)	labs	test_name	Interleukin 1 beta (IL-1B)	result_unit	pg/ml	test_result
Cytokines	Interleukin 10 (IL-10)	labs	test_name	Interleukin 10 (IL-10)	result_unit	pg/ml	test_result
Cytokines	Interleukin 10 (IL-10)	labs	test_name	Interleukin 10 (IL-10)	result_unit		test_result
Cytokines	interleukin 12 (IL-12)	labs	test_name	il-12 (interleukin 12)	result_unit	pg/ml	test_result
Cytokines	interleukin 12 (IL-12)	labs	test_name	interleukin 12	result_unit	pg/ml	test_result
Cytokines	Interleukin 13 (IL-13)	labs	test_name	Interleukin 13 (IL-13)	result_unit	pg/ml	test_result
Cytokines	Interleukin 17 (IL-17)	labs	test_name	Interleukin 17 (IL-17)	result_unit	pg/ml	test_result
Cytokines	interleukin 2 (IL-2)	labs	test_name	il-2 (interleukin 2)	result_unit	pg/ml	test_result
Cytokines	interleukin 2 (IL-2)	labs	test_name	interleukin 2	result_unit	pg/ml	test_result
Cytokines	Interleukin 2 receptor alpha.soluble (CD25)	labs	test_name	Interleukin 2 receptor alpha.soluble (CD25)	result_unit	pg/ml	test_result
Cytokines	Interleukin 2 receptor alpha.soluble (CD25)	labs	test_name	Interleukin 2 receptor alpha.soluble (CD25)	result_unit	u/ml	test_result
Cytokines	Interleukin 4 (IL-4)	labs	test_name	Interleukin 4 (IL-4)	result_unit	pg/ml	test_result
Cytokines	Interleukin 5 (IL-5)	labs	test_name	Interleukin 5 (IL-5)	result_unit	pg/ml	test_result
Cytokines	Interleukin 6 (IL-6)	labs	test_name	Interleukin 6 (IL-6)	result_unit	pg/ml	test_result
Cytokines	Interleukin 6 (IL-6)	labs	test_name	Interleukin 6 (IL-6)	result_unit	underscore	test_result
Cytokines	Interleukin 6 (IL-6)	labs	test_name	Interleukin 6 (IL-6)	result_unit		test_result
Cytokines	Interleukin 8 (IL-8)	labs	test_name	Interleukin 8 (IL-8)	result_unit	pg/ml	test_result
Cytokines	Interleukin 28 b (IL-28b)	labs	test_name	interleukin_28b_interpretation; conversion	result_unit		test_result
Cytokines	Interleukin 28 b (IL-28b)	labs	test_name	interleukin 28b pcr	result_unit		test_result
Cytokines	Transforming growth factor beta (TGF-B)	labs	test_name	Transforming growth factor beta (TGF-B).unspecified spec	result_unit	pg/ml	test_result
Cytokines	Transforming growth factor beta (TGF-B)	labs	test_name	Transforming growth factor beta (TGF-B).unspecified spec	result_unit		test_result
Cytokines	Tumor necrosis factor alpha (TNF-A)	labs	test_name	Tumor necrosis factor.alpha (TNF-A)	result_unit	pg/ml	test_result
Cytokines	Interferon gamma (IFNγ) background	labs	test_name	Gamma interferon background	result_unit	u/ml	test_result
Cytokines	Interferon gamma (IFNγ) background	labs	test_name	Gamma interferon background	result_unit		test_result
Cytokines	Interferon gamma (IFNγ) background	labs	test_name	Gamma interferon background	result_unit	iuml	test_result
Cytokines	Interferon gamma (IFNγ) specific test	labs	test_name	Interferon gamma (IFNγ)	result_unit	pg/ml	test_result
Liver	Triglycerides (TG)	labs	test_name	Triglycerides (TG)	result_unit	mg/dl	test_result
BloodCells	Neutrophil.absolute	labs	test_name	Neutrophil.absolute	result_unit	x10^3/ul	test_result
BloodCells	Neutrophil.absolute	labs	test_name	Neutrophil.absolute	result_unit	thousands/l	test_result
BloodCells	Neutrophil.absolute	labs	test_name	Neutrophil.absolute	result_unit		test_result
BloodCells	Neutrophil.percent	labs	test_name	Neutrophil.percent	result_unit	%	test_result
GeneralLabs	Hemoglobin HGB	labs	test_name	Hemoglobin (HGB)	result_unit	g/dl	test_result
GeneralLabs	Hemoglobin A1C	labs	test_name	Hemoglobin A1C	result_unit	%	test_result
GeneralLabs	Hemoglobin A1C	labs	test_name	Hemoglobin A1C	result_unit	g/dl	test_result
GeneralLabs	Hemoglobin F.percent	labs	test_name	Hemoglobin F.percent	result_unit	%	test_result
GeneralLabs	Mean corpuscular hemoglobin (MCH)	labs	test_name	Mean corpuscular hemoglobin (MCH)	result_unit	pg	test_result
GeneralLabs	Mean corpuscular hemoglobin concentration (MCHC	labs	test_name	Mean corpuscular hemoglobin concentration (MCHC)	result_unit	g/dl	test_result
GeneralLabs	Platelets	labs	test_name	platelets	result_unit	x10^3/ul	test_result
GeneralLabs	Platelets	labs	test_name	Platelets	result_unit		test_result
Hypoxia	Fraction of inspired oxygen (FiO2) no unit	labs	test_name	Fraction of inspired oxygen (FiO2) and/or O2 flow rate.inh	result_unit		test_result
Hypoxia	Fraction of inspired oxygen (FiO2) flow	labs	test_name	Fraction of inspired oxygen (FiO2) and/or O2 flow rate.inh	result_unit	l/min	test_result
Hypoxia	Fraction of inspired oxygen (FiO2) percent	labs	test_name	Fraction of inspired oxygen (FiO2) and/or O2 flow rate.inh	result_unit	%	test_result
Hypoxia	Fraction of inspired oxygen (FiO2) percent	labs	test_name	Fraction of inspired oxygen (FiO2).inhaled gas.percent	result_unit	%	test_result
Hypoxia	Oxygen saturation (SO2).arterial blood	labs	test_name	Oxygen saturation (SO2).arterial blood	result_unit	%	test_result
Hypoxia	Oxygen saturation (SpO2).pulse oximetry	labs	test_name	Oxygen saturation (SpO2).pulse oximetry	result_unit	%	test_result
Hypoxia	oxygen delivery	labs	test_name	oxygen delivery	result_unit		test_result
Hypoxia	oxygen device	labs	test_name	oxygen device	result_unit		test_result
Hypoxia	oxygen therapy	labs	test_name	oxygen therapy	result_unit		test_result
Hypoxia	Oxygen.partial pressure (PO2).arterial blood	labs	test_name	Oxygen.partial pressure (PO2).arterial blood	result_unit	mm Hg	test_result
Hypoxia	Oxygen.partial pressure (PvO2).mixed venous blood	labs	test_name	Oxygen.partial pressure (PvO2).mixed venous blood	result_unit	mm Hg	test_result
Hypoxia	Carbon dioxide.partial pressure (PCO2).arterial blooc	labs	test_name	Carbon dioxide.partial pressure (PCO2).arterial blood	result_unit	mm Hg	test_result
Hypoxia	Carbon dioxide.total (CO2)	labs	test_name	Carbon dioxide.total (CO2)	result_unit	mmol/l	test_result
Hypoxia	Carbon dioxide.total (CO2).arterial blood	labs	test_name	Carbon dioxide.total (CO2).arterial blood	result_unit	mmol/l	test_result
GeneralLabs	Bicarbonate (HCO3)	labs	test_name	Bicarbonate (HCO3)	result_unit	mmol/l	test_result
GeneralLabs	Bicarbonate (HCO3).arterial blood	labs	test_name	Bicarbonate (HCO3).arterial blood	result_unit	mmol/l	test_result

GeneralLabs	Bilirubin.direct	labs	test_name	Bilirubin.direct		result_unit	mg/dl	test_result
GeneralLabs	Bilirubin.total	labs	test_name	Bilirubin.total		result_unit	mg/dl	test_result
GeneralLabs	Bilirubin.transcutaneous	labs	test_name	Bilirubin.transcutaneous		result_unit	mg/dl	test_result
GeneralLabs	Bilirubin.urine.dipstick	labs	test_name	Bilirubin.urine.dipstick		result_unit		test_result
GeneralLabs	Bilirubin.urine.dipstick	labs	test_name	Bilirubin.urine.dipstick		result_unit	mg/dl	test_result
GeneralLabs	Bilirubin.urine.dipstick	labs	test_name	Bilirubin.urine.dipstick		result_unit	units	test_result
GeneralLabs	Calcium.ionized	labs	test_name	Calcium.ionized		result_unit	mg/dl	test_result
GeneralLabs	Calcium.total	labs	test_name	Calcium.total		result_unit	mg/dl	test_result
Temperature	Body temperature	labs	test_name	Body temperature		result_unit		test_result
Temperature	Body temperature	labs	test_name	Body temperature		result_unit	deg-f	test_result
Temperature	Body temperature	labs	test_name	Body temperature		result_unit	deg c	test_result
GeneralLabs	Troponin (TNT) (TNI)	labs	test_name	Troponin (TNT) (TNI)		result_unit	ng/ml	test_result
GeneralLabs	Troponin (TNT) (TNI)	labs	test_name	Troponin (TNT) (TNI)		result_unit		test_result
GeneralLabs	Troponin.I (TNI)	labs	test_name	Troponin.I (TNI)		result_unit	ng/ml	test_result
GeneralLabs	Troponin.I (TNI)	labs	test_name	Troponin.I (TNI)		result_unit		test_result
GeneralLabs	Troponin.T (TNT)	labs	test_name	Troponin.T (TNT)		result_unit	ng/ml	test_result
GeneralLabs	Troponin.T (TNT)	labs	test_name	Troponin.T (TNT)		result_unit		test_result
Covid	Sars-Cov2-Ct-target1-ORF	labs	test_name	sars-cov-2 target 1 cycle threshold		result_unit		test_result
Covid	Sars-Cov2-Ct-target2-any-betacov	labs	test_name	sars-cov-2 target 2 cycle threshold		result_unit		test_result
BloodCells	Lymphocyte.percent	labs	test_name	Lymphocyte.percent		result_unit	%	test_result
BloodCells	Basophil.absolute	labs	test_name	Basophil.absolute		result_unit	x10^3/ul	test_result
BloodCells	Basophil.absolute	labs	test_name	Basophil.absolute		result_unit	thousands/l	test_result
BloodCells	Basophil.absolute	labs	test_name	Basophil.absolute		result_unit		test_result
BloodCells	Basophil.percent	labs	test_name	Basophil.percent		result_unit	%	test_result
BloodCells	Fibrinogen	labs	test_name	Fibrinogen		result_unit	mg/dl	test_result
Inflammation	Fibrin D-Dimer (FEU)	labs	test_name	Fibrin D-Dimer		result_unit	g/ml feu	test_result
Inflammation	Fibrin D-Dimer (FEU)	labs	test_name	Fibrin D-Dimer		result_unit	g/ml feu	test_result
Inflammation	Fibrin D-Dimer (FEU)	labs	test_name	Fibrin D-Dimer		result_unit	mg/ml feu	test_result
Inflammation	Fibrin D-Dimer (FEU)	labs	test_name	Fibrin D-Dimer		result_unit	feu mcg/ml	test_result
Inflammation	Fibrin D-Dimer (FEU)	labs	test_name	Fibrin D-Dimer		result_unit	feu mcg/ml	test_result
Inflammation	Fibrin D-Dimer (FEU)	labs	test_name	Fibrin D-Dimer		result_unit	feu ug/ml	test_result
Inflammation	Fibrin D-Dimer (FEU)	labs	test_name	Fibrin D-Dimer		result_unit	feu ug/ml	test_result
Inflammation	Fibrin D-Dimer (FEU)	labs	test_name	Fibrin D-Dimer		result_unit	mcg/ml feu	test_result
Inflammation	Fibrin D-Dimer (FEU)	labs	test_name	Fibrin D-Dimer		result_unit	mcg/ml feu	test_result
Inflammation	Fibrin D-Dimer (FEU)	labs	test_name	Fibrin D-Dimer		result_unit	mcgfibrinoger	test_result
Inflammation	Fibrin D-Dimer (FEU)	labs	test_name	Fibrin D-Dimer		result_unit	mcgfibrinoger	test_result
Inflammation	Fibrin D-Dimer (FEU)	labs	test_name	Fibrin D-Dimer		result_unit	mg/l (feu)	test_result
Inflammation	Fibrin D-Dimer (FEU)	labs	test_name	Fibrin D-Dimer		result_unit	mg/l (feu)	test_result
Inflammation	Fibrin D-Dimer (FEU)	labs	test_name	Fibrin D-Dimer		result_unit	mg/l(feu)	test_result
Inflammation	Fibrin D-Dimer (FEU)	labs	test_name	Fibrin D-Dimer		result_unit	mg/l(feu)	test_result
Inflammation	Fibrin D-Dimer (FEU)	labs	test_name	Fibrin D-Dimer		result_unit	mg/l[feu]	test_result
Inflammation	Fibrin D-Dimer (FEU)	labs	test_name	Fibrin D-Dimer		result_unit	mg/lfeu	test_result
Inflammation	Fibrin D-Dimer (FEU)	labs	test_name	Fibrin D-Dimer		result_unit	micrograms/r	test_result
Inflammation	Fibrin D-Dimer (FEU)	labs	test_name	Fibrin D-Dimer		result_unit	micrograms/r	test_result
Inflammation	Fibrin D-Dimer (FEU)	labs	test_name	Fibrin D-Dimer		result_unit	milligrams pe	test_result
Inflammation	Fibrin D-Dimer (FEU)	labs	test_name	Fibrin D-Dimer		result_unit	milligrams pe	test_result
Inflammation	Fibrin D-Dimer (FEU)	labs	test_name	Fibrin D-Dimer		result_unit	ug/ml (feu)	test_result
Inflammation	Fibrin D-Dimer (FEU)	labs	test_name	Fibrin D-Dimer		result_unit	ug/ml (feu)	test_result
Inflammation	Fibrin D-Dimer (FEU)	labs	test_name	Fibrin D-Dimer		result_unit	ug/ml feu	test_result
Inflammation	Fibrin D-Dimer (FEU)	labs	test_name	Fibrin D-Dimer		result_unit	ug/ml feu	test_result
Inflammation	Fibrin D-Dimer (FEU)	labs	test_name	Fibrin D-Dimer		result_unit	ug/ml(feu)	test_result
Inflammation	Fibrin D-Dimer (FEU)	labs	test_name	Fibrin D-Dimer		result_unit	ug/ml(feu)	test_result
Inflammation	Fibrin D-Dimer (FEU)	labs	test_name	Fibrin D-Dimer		result_unit	ug/ml[feu]	test_result
Inflammation	Fibrin D-Dimer (DDU)	labs	test_name	Fibrin D-Dimer		result_unit	d-du ng/ml	test_result
Inflammation	Fibrin D-Dimer (DDU)	labs	test_name	Fibrin D-Dimer		result_unit	d-du ng/ml	test_result
Inflammation	Fibrin D-Dimer (DDU)	labs	test_name	Fibrin D-Dimer		result_unit	nanograms/m	test_result
Inflammation	Fibrin D-Dimer (DDU)	labs	test_name	Fibrin D-Dimer		result_unit	nanograms/m	test_result
Inflammation	Fibrin D-Dimer (DDU)	labs	test_name	Fibrin D-Dimer		result_unit	ng/ml (d-du)	test_result
Inflammation	Fibrin D-Dimer (DDU)	labs	test_name	Fibrin D-Dimer		result_unit	ng/ml (d-du)	test_result
Inflammation	Fibrin D-Dimer (DDU)	labs	test_name	Fibrin D-Dimer		result_unit	ng/ml d-dime	test_result
Inflammation	Fibrin D-Dimer (DDU)	labs	test_name	Fibrin D-Dimer		result_unit	ng/ml d-dime	test_result
Inflammation	Fibrin D-Dimer (DDU)	labs	test_name	Fibrin D-Dimer		result_unit	ng/ml d-dime	test_result
Inflammation	Fibrin D-Dimer (DDU)	labs	test_name	Fibrin D-Dimer		result_unit	ng/ml ddu	test_result
Inflammation	Fibrin D-Dimer (DDU)	labs	test_name	Fibrin D-Dimer		result_unit	ng/ml ddu	test_result
Inflammation	Fibrin D-Dimer (DDU)	labs	test_name	Fibrin D-Dimer		result_unit	ng/ml d-du	test_result
Inflammation	Fibrin D-Dimer (DDU)	labs	test_name	Fibrin D-Dimer		result_unit	ng/ml d-du	test_result
Inflammation	Fibrin D-Dimer (DDU)	labs	test_name	Fibrin D-Dimer		result_unit	ng/ml(ddu)	test_result
Inflammation	Fibrin D-Dimer (DDU)	labs	test_name	Fibrin D-Dimer		result_unit	ng/ml(ddu)	test_result
Inflammation	Fibrin D-Dimer (DDU)	labs	test_name	Fibrin D-Dimer		result_unit	ng/ml(d-du)	test_result
Inflammation	Fibrin D-Dimer (DDU)	labs	test_name	Fibrin D-Dimer		result_unit	ng/ml(d-du)	test_result
Inflammation	Fibrin D-Dimer (FEU)	labs	test_name	Fibrin D-Dimer		result_unit	feung/ml	test_result
Inflammation	Fibrin D-Dimer (FEU)	labs	test_name	Fibrin D-Dimer		result_unit	feung/ml	test_result
Inflammation	Fibrin D-Dimer (FEU)	labs	test_name	Fibrin D-Dimer		result_unit	nanograms fe	test_result
Inflammation	Fibrin D-Dimer (FEU)	labs	test_name	Fibrin D-Dimer		result_unit	nanograms fe	test_result
Inflammation	Fibrin D-Dimer (FEU)	labs	test_name	Fibrin D-Dimer		result_unit	nanograms fe	test_result
Inflammation	Fibrin D-Dimer (FEU)	labs	test_name	Fibrin D-Dimer		result_unit	nanograms fe	test_result
Inflammation	Fibrin D-Dimer (FEU)	labs	test_name	Fibrin D-Dimer		result_unit	ng/ml(feu)	test_result
Inflammation	Fibrin D-Dimer (FEU)	labs	test_name	Fibrin D-Dimer		result_unit	ng/ml(feu)	test_result
Inflammation	Fibrin D-Dimer (FEU)	labs	test_name	Fibrin D-Dimer		result_unit	ng/mlfeu	test_result
Inflammation	Fibrin D-Dimer (FEU)	labs	test_name	Fibrin D-Dimer		result_unit	ng/mlfeu	test_result
Inflammation	Fibrin D-Dimer unclear type	labs	test_name	Fibrin D-Dimer		result_unit	mg/l	test_result
Inflammation	Fibrin D-Dimer unclear type	labs	test_name	Fibrin D-Dimer		result_unit	mg/l	test_result
Inflammation	Fibrin D-Dimer unclear type	labs	test_name	Fibrin D-Dimer		result_unit	ng/ml	test_result
Inflammation	Fibrin D-Dimer unclear type	labs	test_name	Fibrin D-Dimer		result_unit	ng/ml	test_result
Inflammation	Fibrin D-Dimer unclear type	labs	test_name	Fibrin D-Dimer		result_unit	ug/ml	test_result
Inflammation	Fibrin D-Dimer unclear type	labs	test_name	Fibrin D-Dimer		result_unit	ug/ml	test_result
vasopressors	amantadine	patient_report	ndc		68084061101			
vasopressors	amantadine	patient_report	ndc		72888003301			
vasopressors	amantadine	patient_report	ndc		68084061111			
vasopressors	amantadine	patient_report	ndc		51079024720			
vasopressors	amantadine	patient_report	ndc		50268006915			
vasopressors	amantadine	patient_report	ndc		62332024631			
vasopressors	amantadine	patient_report	ndc		99999000392			
vasopressors	amantadine	patient_report	ndc		781204801			
vasopressors	amantadine	patient_report	ndc		591493001			
vasopressors	amantadine	patient_report	ndc		121064610			
vasopressors	amantadine	patient_report	ndc		69452014230			
vasopressors	amantadine	patient_report	ndc		832011150			
vasopressors	amantadine	patient_report	ndc		591492001			

vasopressors	amantadine	patient_report ndc	51079048120
vasopressors	amantadine	patient_report ndc	832101500
vasopressors	amantadine	patient_report ndc	69097092512
vasopressors	amantadine	patient_report ndc	10888500602
vasopressors	amantadine	patient_report ndc	60432009316
vasopressors	amantadine	patient_report ndc	50383080716
vasopressors	amantadine	patient_report ndc	73152007505
vasopressors	amantadine	patient_report ndc	69387010005
vasopressors	amantadine	patient_report ndc	73152007605
vasopressors	amantadine	patient_report ndc	121064616
vasopressors	amantadine	patient_report ndc	527170401
vasopressors	amantadine	patient_report ndc	62332058631
vasopressors	amantadine	patient_report ndc	832011100
vasopressors	amantadine	patient_report ndc	69452014220
vasopressors	amantadine	patient_report ndc	75840010301
vasopressors	apomorphine	patient_report ndc	27505000405
vasopressors	apomorphine	patient_report ndc	27505000401
vasopressors	bromocriptine	patient_report ndc	781532531
vasopressors	bromocriptine	patient_report ndc	60687028621
vasopressors	bromocriptine	patient_report ndc	68382011006
vasopressors	bromocriptine	patient_report ndc	63304096230
vasopressors	bromocriptine	patient_report ndc	781181701
vasopressors	bromocriptine	patient_report ndc	378204201
vasopressors	bromocriptine	patient_report ndc	63304096201
vasopressors	bromocriptine	patient_report ndc	574010601
vasopressors	bromocriptine	patient_report ndc	54868566702
vasopressors	bromocriptine	patient_report ndc	574010603
vasopressors	bromocriptine	patient_report ndc	30698001701
vasopressors	bromocriptine	patient_report ndc	378709601
vasopressors	bromocriptine	patient_report ndc	378204293
vasopressors	bromocriptine	patient_report ndc	781532501
vasopressors	bromocriptine	patient_report ndc	378709693
vasopressors	cabergoline	patient_report ndc	93542088
vasopressors	cabergoline	patient_report ndc	60505259702
vasopressors	cabergoline	patient_report ndc	59762100501
vasopressors	cabergoline	patient_report ndc	49884067314
vasopressors	cabergoline	patient_report ndc	50742011808
vasopressors	cabergoline	patient_report ndc	13700112
vasopressors	cabergoline	patient_report ndc	378280026
vasopressors	cabergoline	patient_report ndc	68084024521
vasopressors	desmopressin	patient_report ndc	591246501
vasopressors	desmopressin	patient_report ndc	70860045401
vasopressors	desmopressin	patient_report ndc	69918090110
vasopressors	desmopressin	patient_report ndc	69918089910
vasopressors	desmopressin	patient_report ndc	99999022203
vasopressors	desmopressin	patient_report ndc	70860045410
vasopressors	desmopressin	patient_report ndc	88888100416
vasopressors	desmopressin	patient_report ndc	69918020101
vasopressors	desmopressin	patient_report ndc	591246401
vasopressors	desmopressin	patient_report ndc	93731701
vasopressors	desmopressin	patient_report ndc	68084060621
vasopressors	desmopressin	patient_report ndc	55566230000
vasopressors	desmopressin	patient_report ndc	69918010101
vasopressors	desmopressin	patient_report ndc	62756016191
vasopressors	desmopressin	patient_report ndc	68084060421
vasopressors	desmopressin	patient_report ndc	68382038401
vasopressors	desmopressin	patient_report ndc	60505025801
vasopressors	desmopressin	patient_report ndc	115164601
vasopressors	desmopressin	patient_report ndc	69918050105
vasopressors	desmopressin	patient_report ndc	24208034205
vasopressors	desmopressin	patient_report ndc	60505081500
vasopressors	desmopressin	patient_report ndc	62756052940
vasopressors	desmopressin	patient_report ndc	68462027601
vasopressors	desmopressin	patient_report ndc	66993002501
vasopressors	desmopressin	patient_report ndc	93731601
vasopressors	Dobutamine	patient_report ndc	55390056090
vasopressors	Dobutamine	patient_report ndc	409234732
vasopressors	Dobutamine	patient_report ndc	55390056099
vasopressors	Dobutamine	patient_report ndc	99999900309
vasopressors	Dobutamine	patient_report ndc	99999999947
vasopressors	Dobutamine	patient_report ndc	88888100496
vasopressors	Dobutamine	patient_report ndc	338107502
vasopressors	Dobutamine	patient_report ndc	409234632
vasopressors	Dobutamine	patient_report ndc	1003040923
vasopressors	Dobutamine	patient_report ndc	11994001104
vasopressors	Dobutamine	patient_report ndc	338107302
vasopressors	Dobutamine	patient_report ndc	409372432
vasopressors	Dobutamine	patient_report ndc	338107702
vasopressors	Dobutamine	patient_report ndc	409234488
vasopressors	Dobutamine	patient_report ndc	409234401
vasopressors	Dobutamine	patient_report ndc	409202520
vasopressors	Dobutamine	patient_report ndc	409202554
vasopressors	Dopamine	patient_report ndc	517190525
vasopressors	Dopamine	patient_report ndc	517180525
vasopressors	Dopamine	patient_report ndc	590004006
vasopressors	Dopamine	patient_report ndc	409910420
vasopressors	Dopamine	patient_report ndc	338100702
vasopressors	Dopamine	patient_report ndc	409780922
vasopressors	Dopamine	patient_report ndc	99999900398
vasopressors	Dopamine	patient_report ndc	99999999950
vasopressors	Dopamine	patient_report ndc	88888100497
vasopressors	Dopamine	patient_report ndc	99999071521
vasopressors	Dopamine	patient_report ndc	51927231900
vasopressors	Dopamine	patient_report ndc	409582001
vasopressors	Dopamine	patient_report ndc	143925201
vasopressors	Dopamine	patient_report ndc	409910421
vasopressors	entacapone	patient_report ndc	64679078102
vasopressors	entacapone	patient_report ndc	65862065401
vasopressors	entacapone	patient_report ndc	64679078604

vasopressors	entacapone	patient_report ndc	27241004910
vasopressors	entacapone	patient_report ndc	60687018821
vasopressors	entacapone	patient_report ndc	64679078702
vasopressors	entacapone	patient_report ndc	60687018811
vasopressors	entacapone	patient_report ndc	378830101
vasopressors	entacapone	patient_report ndc	378908001
vasopressors	entacapone	patient_report ndc	52427080001
vasopressors	entacapone	patient_report ndc	47335000588
vasopressors	entacapone	patient_report ndc	78032705
vasopressors	entacapone	patient_report ndc	378830201
vasopressors	entacapone	patient_report ndc	47335000788
vasopressors	entacapone	patient_report ndc	64679078704
vasopressors	entacapone	patient_report ndc	64679078404
vasopressors	epinephrine	patient_report ndc	70121157607
vasopressors	epinephrine	patient_report ndc	76329906100
vasopressors	epinephrine	patient_report ndc	63323048927
vasopressors	epinephrine	patient_report ndc	63323046837
vasopressors	epinephrine	patient_report ndc	71019022004
vasopressors	epinephrine	patient_report ndc	71019022207
vasopressors	epinephrine	patient_report ndc	71019022001
vasopressors	epinephrine	patient_report ndc	49502050002
vasopressors	epinephrine	patient_report ndc	487590199
vasopressors	epinephrine	patient_report ndc	17478005002
vasopressors	epinephrine	patient_report ndc	66312017616
vasopressors	epinephrine	patient_report ndc	66975042551
vasopressors	epinephrine	patient_report ndc	55390000210
vasopressors	epinephrine	patient_report ndc	17478005005
vasopressors	epinephrine	patient_report ndc	409337504
vasopressors	epinephrine	patient_report ndc	17478092019
vasopressors	epinephrine	patient_report ndc	61553014848
vasopressors	epinephrine	patient_report ndc	63323046830
vasopressors	epinephrine	patient_report ndc	72196605701
vasopressors	epinephrine	patient_report ndc	10885000210
vasopressors	epinephrine	patient_report ndc	99999999940
vasopressors	epinephrine	patient_report ndc	99999999951
vasopressors	epinephrine	patient_report ndc	76329331601
vasopressors	epinephrine	patient_report ndc	99999999655
vasopressors	epinephrine	patient_report ndc	71019021806
vasopressors	epinephrine	patient_report ndc	88888100516
vasopressors	epinephrine	patient_report ndc	88888300728
vasopressors	epinephrine	patient_report ndc	99999999998
vasopressors	epinephrine	patient_report ndc	77333083110
vasopressors	epinephrine	patient_report ndc	99999001001
vasopressors	epinephrine	patient_report ndc	66467097305
vasopressors	epinephrine	patient_report ndc	70092145336
vasopressors	epinephrine	patient_report ndc	99999990121
vasopressors	epinephrine	patient_report ndc	63323028635
vasopressors	epinephrine	patient_report ndc	99999000347
vasopressors	epinephrine	patient_report ndc	99999900317
vasopressors	epinephrine	patient_report ndc	54288010310
vasopressors	epinephrine	patient_report ndc	63323048327
vasopressors	epinephrine	patient_report ndc	543071012
vasopressors	epinephrine	patient_report ndc	409724101
vasopressors	epinephrine	patient_report ndc	63323046357
vasopressors	epinephrine	patient_report ndc	99999900344
vasopressors	epinephrine	patient_report ndc	76329906000
vasopressors	epinephrine	patient_report ndc	42023015925
vasopressors	epinephrine	patient_report ndc	63323046157
vasopressors	epinephrine	patient_report ndc	78670013102
vasopressors	epinephrine	patient_report ndc	70092161243
vasopressors	epinephrine	patient_report ndc	409317801
vasopressors	epinephrine	patient_report ndc	49502010202
vasopressors	epinephrine	patient_report ndc	703115303
vasopressors	epinephrine	patient_report ndc	63323046217
vasopressors	epinephrine	patient_report ndc	42023016801
vasopressors	epinephrine	patient_report ndc	70092113544
vasopressors	epinephrine	patient_report ndc	93598619
vasopressors	epinephrine	patient_report ndc	54569642500
vasopressors	epinephrine	patient_report ndc	49502010102
vasopressors	epinephrine	patient_report ndc	93598627
vasopressors	epinephrine	patient_report ndc	115169549
vasopressors	epinephrine	patient_report ndc	54505010102
vasopressors	epinephrine	patient_report ndc	115169449
vasopressors	epinephrine	patient_report ndc	93598519
vasopressors	epinephrine	patient_report ndc	54505010202
vasopressors	epinephrine	patient_report ndc	54505010201
vasopressors	epinephrine	patient_report ndc	68788637602
vasopressors	epinephrine	patient_report ndc	409493301
vasopressors	epinephrine	patient_report ndc	62250010310
vasopressors	epinephrine	patient_report ndc	71754000101
vasopressors	epinephrine	patient_report ndc	51552052603
vasopressors	epinephrine	patient_report ndc	38739003001
vasopressors	levodopa	patient_report ndc	228253910
vasopressors	levodopa	patient_report ndc	68084028201
vasopressors	levodopa	patient_report ndc	99999002002
vasopressors	levodopa	patient_report ndc	68084028211
vasopressors	levodopa	patient_report ndc	71205878306
vasopressors	levodopa	patient_report ndc	64679078604
vasopressors	levodopa	patient_report ndc	904623761
vasopressors	levodopa	patient_report ndc	68084009301
vasopressors	levodopa	patient_report ndc	62756051818
vasopressors	levodopa	patient_report ndc	99999999816
vasopressors	levodopa	patient_report ndc	63739001710
vasopressors	levodopa	patient_report ndc	64679078702
vasopressors	levodopa	patient_report ndc	904623861
vasopressors	levodopa	patient_report ndc	68084009311
vasopressors	levodopa	patient_report ndc	68084028101
vasopressors	levodopa	patient_report ndc	93029301
vasopressors	levodopa	patient_report ndc	99999051888

vasopressors	levodopa	patient_report ndc	62756051888
vasopressors	levodopa	patient_report ndc	69367033910
vasopressors	levodopa	patient_report ndc	378830101
vasopressors	levodopa	patient_report ndc	228254010
vasopressors	levodopa	patient_report ndc	62756051788
vasopressors	levodopa	patient_report ndc	63739004610
vasopressors	levodopa	patient_report ndc	63739010810
vasopressors	levodopa	patient_report ndc	68084009411
vasopressors	milrinone	patient_report ndc	63323061720
vasopressors	milrinone	patient_report ndc	60505071800
vasopressors	milrinone	patient_report ndc	63323061710
vasopressors	milrinone	patient_report ndc	99999900453
vasopressors	milrinone	patient_report ndc	71288020021
vasopressors	milrinone	patient_report ndc	99999999964
vasopressors	milrinone	patient_report ndc	63323061750
vasopressors	milrinone	patient_report ndc	88888300301
vasopressors	milrinone	patient_report ndc	338601048
vasopressors	milrinone	patient_report ndc	143932601
vasopressors	milrinone	patient_report ndc	143971901
vasopressors	milrinone	patient_report ndc	71288020011
vasopressors	milrinone	patient_report ndc	409277623
vasopressors	milrinone	patient_report ndc	55390007901
vasopressors	milrinone	patient_report ndc	143971801
vasopressors	milrinone	patient_report ndc	143971810
vasopressors	milrinone	patient_report ndc	55150028710
vasopressors	milrinone	patient_report ndc	143970801
vasopressors	norepinephrine	patient_report ndc	70121157607
vasopressors	norepinephrine	patient_report ndc	71019022004
vasopressors	norepinephrine	patient_report ndc	71019022207
vasopressors	norepinephrine	patient_report ndc	71019022001
vasopressors	norepinephrine	patient_report ndc	55390000210
vasopressors	norepinephrine	patient_report ndc	409337504
vasopressors	norepinephrine	patient_report ndc	72196605701
vasopressors	norepinephrine	patient_report ndc	99999999940
vasopressors	norepinephrine	patient_report ndc	71019021806
vasopressors	norepinephrine	patient_report ndc	99999000347
vasopressors	norepinephrine	patient_report ndc	99999900344
vasopressors	norepinephrine	patient_report ndc	703115303
vasopressors	norepinephrine	patient_report ndc	36000016210
vasopressors	norepinephrine	patient_report ndc	143931801
vasopressors	norepinephrine	patient_report ndc	143931810
vasopressors	phenylephrine	patient_report ndc	76014000433
vasopressors	phenylephrine	patient_report ndc	61314034202
vasopressors	phenylephrine	patient_report ndc	76014000425
vasopressors	phenylephrine	patient_report ndc	99999700681
vasopressors	phenylephrine	patient_report ndc	99999999901
vasopressors	phenylephrine	patient_report ndc	65035905
vasopressors	phenylephrine	patient_report ndc	76014000410
vasopressors	phenylephrine	patient_report ndc	641614225
vasopressors	phenylephrine	patient_report ndc	88888300614
vasopressors	phenylephrine	patient_report ndc	99999000139
vasopressors	phenylephrine	patient_report ndc	69536010015
vasopressors	phenylephrine	patient_report ndc	61314034201
vasopressors	phenylephrine	patient_report ndc	61314035502
vasopressors	phenylephrine	patient_report ndc	42702010305
vasopressors	phenylephrine	patient_report ndc	42002030001
vasopressors	phenylephrine	patient_report ndc	99999001003
vasopressors	phenylephrine	patient_report ndc	69374095710
vasopressors	phenylephrine	patient_report ndc	536129136
vasopressors	phenylephrine	patient_report ndc	87701091667
vasopressors	phenylephrine	patient_report ndc	573286893
vasopressors	phenylephrine	patient_report ndc	99999999044
vasopressors	phenylephrine	patient_report ndc	61553031265
vasopressors	phenylephrine	patient_report ndc	61990021103
vasopressors	phenylephrine	patient_report ndc	71288080702
vasopressors	phenylephrine	patient_report ndc	45802018816
vasopressors	phenylephrine	patient_report ndc	88888300496
vasopressors	phenylephrine	patient_report ndc	66758001604
vasopressors	phenylephrine	patient_report ndc	71449000111
vasopressors	phenylephrine	patient_report ndc	76329630005
vasopressors	phenylephrine	patient_report ndc	71019026307
vasopressors	phenylephrine	patient_report ndc	99999000145
vasopressors	phenylephrine	patient_report ndc	87701041110
vasopressors	phenylephrine	patient_report ndc	99999000089
vasopressors	phenylephrine	patient_report ndc	70069012101
vasopressors	phenylephrine	patient_report ndc	71449000115
vasopressors	phenylephrine	patient_report ndc	17478020605
vasopressors	phenylephrine	patient_report ndc	77777000164
vasopressors	phenylephrine	patient_report ndc	573288320
vasopressors	phenylephrine	patient_report ndc	61553034670
vasopressors	phenylephrine	patient_report ndc	69536005015
vasopressors	phenylephrine	patient_report ndc	54569582000
vasopressors	phenylephrine	patient_report ndc	12830086404
vasopressors	phenylephrine	patient_report ndc	50383080216
vasopressors	phenylephrine	patient_report ndc	58407062530
vasopressors	phenylephrine	patient_report ndc	10267013506
vasopressors	phenylephrine	patient_report ndc	62991204605
vasopressors	phenylephrine	patient_report ndc	12830081616
vasopressors	phenylephrine	patient_report ndc	54569105400
vasopressors	phenylephrine	patient_report ndc	42702010215
vasopressors	phenylephrine	patient_report ndc	66758001701
vasopressors	phenylephrine	patient_report ndc	31871612
vasopressors	phenylephrine	patient_report ndc	51552023205
vasopressors	phenylephrine	patient_report ndc	42192050216
vasopressors	phenylephrine	patient_report ndc	50383080516
vasopressors	phenylephrine	patient_report ndc	50991060716
vasopressors	phenylephrine	patient_report ndc	55289043020
vasopressors	phenylephrine	patient_report ndc	121092516
vasopressors	phenylephrine	patient_report ndc	58605042201

vasopressors	phenylephrine	patient_report	ndc		60258026901	
vasopressors	pramipexole	patient_report	ndc		68462033390	
vasopressors	pramipexole	patient_report	ndc		68462033490	
vasopressors	pramipexole	patient_report	ndc		68462033190	
vasopressors	pramipexole	patient_report	ndc		68462033290	
vasopressors	pramipexole	patient_report	ndc		68462033090	
vasopressors	pramipexole	patient_report	ndc		597018461	
vasopressors	pramipexole	patient_report	ndc		378170705	
vasopressors	pramipexole	patient_report	ndc		378170505	
vasopressors	pramipexole	patient_report	ndc		57237018490	
vasopressors	pramipexole	patient_report	ndc		378170405	
vasopressors	pramipexole	patient_report	ndc		904670461	
vasopressors	pramipexole	patient_report	ndc		378171105	
vasopressors	pramipexole	patient_report	ndc		65862060799	
vasopressors	pramipexole	patient_report	ndc		13668009190	
vasopressors	pramipexole	patient_report	ndc		68382019916	
vasopressors	pramipexole	patient_report	ndc		378171205	
vasopressors	pramipexole	patient_report	ndc		13668009290	
vasopressors	pramipexole	patient_report	ndc		378171377	
vasopressors	ropinirole	patient_report	ndc		68462025601	
vasopressors	ropinirole	patient_report	ndc		68462025501	
vasopressors	ropinirole	patient_report	ndc		68462025901	
vasopressors	ropinirole	patient_report	ndc		68462025801	
vasopressors	ropinirole	patient_report	ndc		68084030621	
vasopressors	ropinirole	patient_report	ndc		68462025701	
vasopressors	ropinirole	patient_report	ndc		68462025401	
vasopressors	ropinirole	patient_report	ndc		68462025301	
vasopressors	ropinirole	patient_report	ndc		62332003231	
vasopressors	ropinirole	patient_report	ndc		54011825	
vasopressors	ropinirole	patient_report	ndc		378550101	
vasopressors	ropinirole	patient_report	ndc		43547026810	
vasopressors	ropinirole	patient_report	ndc		904637461	
vasopressors	ropinirole	patient_report	ndc		378555001	
vasopressors	ropinirole	patient_report	ndc		378552501	
vasopressors	ropinirole	patient_report	ndc		378550201	
vasopressors	ropinirole	patient_report	ndc		43547027010	
vasopressors	ropinirole	patient_report	ndc		904637361	
vasopressors	ropinirole	patient_report	ndc		54011725	
vasopressors	ropinirole	patient_report	ndc		68084031101	
vasopressors	ropinirole	patient_report	ndc		378550401	
vasopressors	ropinirole	patient_report	ndc		378550301	
vasopressors	ropinirole	patient_report	ndc		54011625	
vasopressors	ropinirole	patient_report	ndc		54011925	
vasopressors	rotigotine	patient_report	ndc		50474080403	
vasopressors	rotigotine	patient_report	ndc		50474080603	
vasopressors	rotigotine	patient_report	ndc		99999044004	
vasopressors	rotigotine	patient_report	ndc		50474080203	
vasopressors	vasopressin	patient_report	ndc		63323030201	
vasopressors	vasopressin	patient_report	ndc		42023016425	
vasopressors	vasopressin	patient_report	ndc		42023016410	
vasopressors	vasopressin	patient_report	ndc		77777000185	
systolic	SBP	observations	obs_type	SBP		obs_result
diastolic	DBP	observations	obs_type	DBP		obs_result
temperature	TEMP	observations	obs_type	TEMP		obs_result
ventilation CPAP	CPAP unspecified type	procedure	proc_code		1014859	
ventilation CPAP	CPAP unspecified type	procedure	proc_code		94002	
ventilation CPAP	CPAP unspecified type	procedure	proc_code		94003	
ventilation CPAP	CPAP invasive	procedure	proc_code	5A19		
ventilation lower	ventilation non-mechanical mask	procedure	proc_code	5A19054		
ventilation CPAP	CPAP invasive	procedure	proc_code	5A1945Z		
ventilation CPAP	CPAP invasive	procedure	proc_code	5A1955Z		
ventilation CPAP	CPAP invasive	procedure	proc_code	5A1935Z		
ventilation lower	ventilation non-invasive unspecified type	procedure	proc_code	5A09		
ventilation lower	ventilation high flow cannula	procedure	proc_code	5A0935A		
ventilation lower	ventilation high flow cannula	procedure	proc_code	5A0945A		
ventilation lower	ventilation high flow cannula	procedure	proc_code	5A0955A		
ventilation lower	ventilaiton intermittent positive pressure	procedure	proc_code	5A09358		
ventilation lower	ventilaiton intermittent positive pressure	procedure	proc_code	5A09458		
ventilation lower	ventilaiton intermittent positive pressure	procedure	proc_code	5A09558		
ventilation CPAP	CPAP unspecified type	procedure	proc_code		94660	
ventilation CPAP	CPAP non-invasive	procedure	proc_code	5A09357		
ventilation CPAP	CPAP non-invasive	procedure	proc_code	5A09457		
ventilation CPAP	CPAP non-invasive	procedure	proc_code	5A09557		
ventilation lower	CNAP non-invasive	procedure	proc_code	5A09359		
ventilation lower	CNAP non-invasive	procedure	proc_code	5A09459		
ventilation lower	CNAP non-invasive	procedure	proc_code	5A09559		
ventilation lower	unspecific suspected ventilation	procedure	proc_code	20124-4		
ventilation lower	unspecific suspected ventilation	procedure	proc_code	F024GZZ		
ventilation lower	unspecific suspected ventilation	procedure	proc_code	E0481		
ventilation lower	unspecific suspected ventilation	procedure	proc_code	5A1221J		
ventilation lower	unspecific suspected ventilation	procedure	proc_code	5A1522G		
ventilation lower	unspecific suspected ventilation	procedure	proc_code	5A1945Z		
ventilation lower	unspecific suspected ventilation	procedure	proc_code	5A1D70Z		
ventilation lower	ventilaton intermittent negative pressure	procedure	proc_code	5A0935B		
ventilation lower	ventilaton intermittent negative pressure	procedure	proc_code	5A0945B		
ventilation lower	ventilaton intermittent negative pressure	procedure	proc_code	5A0955B		
intubation	complicated intubation	diagnosis	diagnosis_cd	T884		
intubation	complicated intubation	diagnosis	diagnosis_cd	T888		
intubation	complicated intubation	diagnosis	diagnosis_cd	T884XXA		
intubation	complicated intubation	diagnosis	diagnosis_cd	T884XXD		
intubation	complicated intubation	diagnosis	diagnosis_cd	T884XXS		
intubation	complicated intubation	diagnosis	diagnosis_cd	T8889XA		
ventilation lower	dependence on ventilation	diagnosis	diagnosis_cd	Z990		
ventilation lower	dependence on ventilation	diagnosis	diagnosis_cd	Z991		
ventilation lower	dependence on ventilation	diagnosis	diagnosis_cd	Z9911		
ventilation lower	dependence on ventilation	diagnosis	diagnosis_cd	Z9912		
ventilation lower	dependence on ventilation	diagnosis	diagnosis_cd	Z998		
ventilation lower	dependence on ventilation	diagnosis	diagnosis_cd	Z9981		
ventilation lower	dependence on ventilation	diagnosis	diagnosis_cd	Z9989		

ventilation lower	dependence on ventilation	diagnosis	diagnosis_cd	J9585
intubation	intubation	procedure	proc_code	31500
intubation	intubation	procedure	proc_code	52765003
intubation	intubation	procedure	proc_code	33444-1
intubation	intubation	procedure	proc_code	447996002
intubation	intubation	procedure	proc_code	00718DZ
intubation	intubation	procedure	proc_code	00717DZ
intubation	intubation	procedure	proc_code	0WHQ7YZ
intubation	intubation	procedure	proc_code	0BH07DZ
intubation	intubation	procedure	proc_code	31502
intubation	intubation	procedure	proc_code	0BH17EZ
intubation	intubation	procedure	proc_code	0BH18EZ
intubation	intubation	procedure	proc_code	0B21XEZ
intubation	intubation	procedure	proc_code	112798008
intensive care	intensive care	procedure	proc_code	1013729
intensive care	intensive care	procedure	proc_code	1014309
intensive care	intensive care	procedure	proc_code	99291
intensive care	intensive care	procedure	proc_code	99292
intensive care	intensive care	procedure	proc_code	1019134
intensive care	intensive care	procedure	proc_code	1019130
intensive care	intensive care	procedure	proc_code	99469
intensive care	intensive care	procedure	proc_code	99468
intensive care	intensive care	procedure	proc_code	1019334
intensive care	intensive care	procedure	proc_code	99472
intensive care	intensive care	procedure	proc_code	99471
intensive care	intensive care	procedure	proc_code	99475
intensive care	intensive care	procedure	proc_code	99476
vasopressors	use of vasopressor	procedure	proc_code	7
vasopressors	use of vasopressor	procedure	proc_code	H01BA
vasopressors	use of vasopressor	procedure	proc_code	84588
vasopressors	use of vasopressor	procedure	proc_code	1011327
vasopressors	use of vasopressor	procedure	proc_code	7512
vasopressors	use of vasopressor	procedure	proc_code	8163
vasopressors	use of vasopressor	procedure	proc_code	3992
vasopressors	use of vasopressor	procedure	proc_code	N04BC
vasopressors	use of vasopressor	procedure	proc_code	N04B
vasopressors	use of vasopressor	procedure	proc_code	3628
hypotension	hypotension	diagnosis	diagnosis_cd	I95
hypotension	hypotension	diagnosis	diagnosis_cd	I950
hypotension	hypotension	diagnosis	diagnosis_cd	I951
hypotension	hypotension	diagnosis	diagnosis_cd	I952
hypotension	hypotension	diagnosis	diagnosis_cd	I953
hypotension	hypotension	diagnosis	diagnosis_cd	I958
hypotension	hypotension	diagnosis	diagnosis_cd	I959
hypotension	hypotension	diagnosis	diagnosis_cd	I9581
hypotension	hypotension	diagnosis	diagnosis_cd	I9589
respiratory failure	respiratory failure	diagnosis	diagnosis_cd	J96
respiratory failure	respiratory failure	diagnosis	diagnosis_cd	J960
respiratory failure	respiratory failure	diagnosis	diagnosis_cd	J9600
respiratory failure	respiratory failure	diagnosis	diagnosis_cd	J9601
respiratory failure	respiratory failure	diagnosis	diagnosis_cd	J961
respiratory failure	respiratory failure	diagnosis	diagnosis_cd	J9610
respiratory failure	respiratory failure	diagnosis	diagnosis_cd	J9611
respiratory failure	respiratory failure	diagnosis	diagnosis_cd	J962
respiratory failure	respiratory failure	diagnosis	diagnosis_cd	J9621
respiratory failure	respiratory failure	diagnosis	diagnosis_cd	J9620
respiratory failure	respiratory failure	diagnosis	diagnosis_cd	J9691
covid	covid_diag	diagnosis	diagnosis_cd	U070
covid	covid_diag	diagnosis	diagnosis_cd	J1281
covid	covid_diag	diagnosis	diagnosis_cd	B342
covid	covid_diag	diagnosis	diagnosis_cd	B9729
covid	covid_diag	diagnosis	diagnosis_cd	U072
covid	covid_diag	diagnosis	diagnosis_cd	U07
covid	covid_diag	diagnosis	diagnosis_cd	U071
covid	covid_diag	diagnosis	diagnosis_cd	J1282
sepsis	sepsis_patho_diag	diagnosis	diagnosis_cd	A40
sepsis	sepsis_patho_diag	diagnosis	diagnosis_cd	A400
sepsis	sepsis_patho_diag	diagnosis	diagnosis_cd	A401
sepsis	sepsis_patho_diag	diagnosis	diagnosis_cd	A403
sepsis	sepsis_patho_diag	diagnosis	diagnosis_cd	A408
sepsis	sepsis_patho_diag	diagnosis	diagnosis_cd	A409
sepsis	sepsis_patho_diag	diagnosis	diagnosis_cd	A41
sepsis	sepsis_patho_diag	diagnosis	diagnosis_cd	A410
sepsis	sepsis_patho_diag	diagnosis	diagnosis_cd	A4101
sepsis	sepsis_patho_diag	diagnosis	diagnosis_cd	A4102
sepsis	sepsis_patho_diag	diagnosis	diagnosis_cd	A411
sepsis	sepsis_patho_diag	diagnosis	diagnosis_cd	A412
sepsis	sepsis_patho_diag	diagnosis	diagnosis_cd	A413
sepsis	sepsis_patho_diag	diagnosis	diagnosis_cd	A414
sepsis	sepsis_patho_diag	diagnosis	diagnosis_cd	A415
sepsis	sepsis_patho_diag	diagnosis	diagnosis_cd	A4150
sepsis	sepsis_patho_diag	diagnosis	diagnosis_cd	A4151
sepsis	sepsis_patho_diag	diagnosis	diagnosis_cd	A4152
sepsis	sepsis_patho_diag	diagnosis	diagnosis_cd	A4153
sepsis	sepsis_patho_diag	diagnosis	diagnosis_cd	A4159
sepsis	sepsis_patho_diag	diagnosis	diagnosis_cd	A418
sepsis	sepsis_patho_diag	diagnosis	diagnosis_cd	A4181
sepsis	sepsis_patho_diag	diagnosis	diagnosis_cd	A4189
sepsis	sepsis_patho_diag	diagnosis	diagnosis_cd	A419
Hemochromatosis	Hemochromatosis	diagnosis	diagnosis_cd	E83118
Hemochromatosis	Hemochromatosis	diagnosis	diagnosis_cd	E83110
Hemochromatosis	Hemochromatosis	diagnosis	diagnosis_cd	E83111
Hemochromatosis	Hemochromatosis	diagnosis	diagnosis_cd	E8311
Hemochromatosis	Hemochromatosis	diagnosis	diagnosis_cd	E8311
Hemochromatosis	Hemochromatosis	diagnosis	diagnosis_cd	E83119
neutropenia	neutropenia	diagnosis	diagnosis_cd	D70
neutropenia	neutropenia	diagnosis	diagnosis_cd	D700
neutropenia	neutropenia	diagnosis	diagnosis_cd	D701
neutropenia	neutropenia	diagnosis	diagnosis_cd	D702

neutropenia	neutropenia	diagnosis	diagnosis_cd	D703
neutropenia	neutropenia	diagnosis	diagnosis_cd	D704
neutropenia	neutropenia	diagnosis	diagnosis_cd	D708
neutropenia	neutropenia	diagnosis	diagnosis_cd	D709
potential neutropenic fev	fever not classified (neutropenic)	diagnosis	diagnosis_cd	R5081
Stills disease	Adult onset Still's disease	diagnosis	diagnosis_cd	M061
potential HLH	histiocytosis	diagnosis	diagnosis_cd	D761
potential HLH	histiocytosis	diagnosis	diagnosis_cd	D762
potential HLH	histiocytosis	diagnosis	diagnosis_cd	D763
Castlemans disease	Castlemans disease	diagnosis	diagnosis_cd	D47Z2
MIS	Multisystemic inflammatory syndrome	diagnosis	diagnosis_cd	M3581
potential sJIA	Juvenile (idiopathic) arthritis	diagnosis	diagnosis_cd	M08461
potential sJIA	Juvenile (idiopathic) arthritis	diagnosis	diagnosis_cd	M0840
potential sJIA	Juvenile (idiopathic) arthritis	diagnosis	diagnosis_cd	M0809
potential sJIA	Juvenile (idiopathic) arthritis	diagnosis	diagnosis_cd	M08022
potential sJIA	Juvenile (idiopathic) arthritis	diagnosis	diagnosis_cd	M08211
potential sJIA	Juvenile (idiopathic) arthritis	diagnosis	diagnosis_cd	M0890
potential sJIA	Juvenile (idiopathic) arthritis	diagnosis	diagnosis_cd	M08072
potential sJIA	Juvenile (idiopathic) arthritis	diagnosis	diagnosis_cd	M08852
potential sJIA	Juvenile (idiopathic) arthritis	diagnosis	diagnosis_cd	M0880
potential sJIA	Juvenile (idiopathic) arthritis	diagnosis	diagnosis_cd	M08979
potential sJIA	Juvenile (idiopathic) arthritis	diagnosis	diagnosis_cd	M08961
potential sJIA	Juvenile (idiopathic) arthritis	diagnosis	diagnosis_cd	M08861
potential sJIA	Juvenile (idiopathic) arthritis	diagnosis	diagnosis_cd	M0828
potential sJIA	Juvenile (idiopathic) arthritis	diagnosis	diagnosis_cd	M08822
potential sJIA	Juvenile (idiopathic) arthritis	diagnosis	diagnosis_cd	M08041
potential sJIA	Juvenile (idiopathic) arthritis	diagnosis	diagnosis_cd	M08032
potential sJIA	Juvenile (idiopathic) arthritis	diagnosis	diagnosis_cd	M08071
potential sJIA	Juvenile (idiopathic) arthritis	diagnosis	diagnosis_cd	M08479
potential sJIA	Juvenile (idiopathic) arthritis	diagnosis	diagnosis_cd	M08051
potential sJIA	Juvenile (idiopathic) arthritis	diagnosis	diagnosis_cd	M08279
potential sJIA	Juvenile (idiopathic) arthritis	diagnosis	diagnosis_cd	M08012
potential sJIA	Juvenile (idiopathic) arthritis	diagnosis	diagnosis_cd	M08069
potential sJIA	Juvenile (idiopathic) arthritis	diagnosis	diagnosis_cd	M08462
potential sJIA	Juvenile (idiopathic) arthritis	diagnosis	diagnosis_cd	M0898
potential sJIA	Juvenile (idiopathic) arthritis	diagnosis	diagnosis_cd	M08931
potential sJIA	Juvenile (idiopathic) arthritis	diagnosis	diagnosis_cd	M08062
potential sJIA	Juvenile (idiopathic) arthritis	diagnosis	diagnosis_cd	M08272
potential sJIA	Juvenile (idiopathic) arthritis	diagnosis	diagnosis_cd	M08859
potential sJIA	Juvenile (idiopathic) arthritis	diagnosis	diagnosis_cd	M08922
potential sJIA	Juvenile (idiopathic) arthritis	diagnosis	diagnosis_cd	M08229
potential sJIA	Juvenile (idiopathic) arthritis	diagnosis	diagnosis_cd	M08871
potential sJIA	Juvenile (idiopathic) arthritis	diagnosis	diagnosis_cd	M0808
potential sJIA	Juvenile (idiopathic) arthritis	diagnosis	diagnosis_cd	M0899
potential sJIA	Juvenile (idiopathic) arthritis	diagnosis	diagnosis_cd	M08042
potential sJIA	Juvenile (idiopathic) arthritis	diagnosis	diagnosis_cd	M082A
potential sJIA	Juvenile (idiopathic) arthritis	diagnosis	diagnosis_cd	M08059
potential sJIA	Juvenile (idiopathic) arthritis	diagnosis	diagnosis_cd	M0889
potential sJIA	Juvenile (idiopathic) arthritis	diagnosis	diagnosis_cd	M08021
potential sJIA	Juvenile (idiopathic) arthritis	diagnosis	diagnosis_cd	M08932
potential sJIA	Juvenile (idiopathic) arthritis	diagnosis	diagnosis_cd	M081
potential sJIA	Juvenile (idiopathic) arthritis	diagnosis	diagnosis_cd	M08241
potential sJIA	Juvenile (idiopathic) arthritis	diagnosis	diagnosis_cd	M08952
potential sJIA	Juvenile (idiopathic) arthritis	diagnosis	diagnosis_cd	M080A
potential sJIA	Juvenile (idiopathic) arthritis	diagnosis	diagnosis_cd	M08412
potential sJIA	Juvenile (idiopathic) arthritis	diagnosis	diagnosis_cd	M08959
potential sJIA	Juvenile (idiopathic) arthritis	diagnosis	diagnosis_cd	M08472
potential sJIA	Juvenile (idiopathic) arthritis	diagnosis	diagnosis_cd	M08422
potential sJIA	Juvenile (idiopathic) arthritis	diagnosis	diagnosis_cd	M08941
potential sJIA	Juvenile (idiopathic) arthritis	diagnosis	diagnosis_cd	M08911
potential sJIA	Juvenile (idiopathic) arthritis	diagnosis	diagnosis_cd	M08839
potential sJIA	Juvenile (idiopathic) arthritis	diagnosis	diagnosis_cd	M08842
potential sJIA	Juvenile (idiopathic) arthritis	diagnosis	diagnosis_cd	M08862
potential sJIA	Juvenile (idiopathic) arthritis	diagnosis	diagnosis_cd	M084A
potential sJIA	Juvenile (idiopathic) arthritis	diagnosis	diagnosis_cd	M08869
potential sJIA	Juvenile (idiopathic) arthritis	diagnosis	diagnosis_cd	M08969
potential sJIA	Juvenile (idiopathic) arthritis	diagnosis	diagnosis_cd	M08949
potential sJIA	Juvenile (idiopathic) arthritis	diagnosis	diagnosis_cd	M08451
potential sJIA	Juvenile (idiopathic) arthritis	diagnosis	diagnosis_cd	M08912
potential sJIA	Juvenile (idiopathic) arthritis	diagnosis	diagnosis_cd	M08232
potential sJIA	Juvenile (idiopathic) arthritis	diagnosis	diagnosis_cd	M08921
potential sJIA	Juvenile (idiopathic) arthritis	diagnosis	diagnosis_cd	M08929
potential sJIA	Juvenile (idiopathic) arthritis	diagnosis	diagnosis_cd	M08469
potential sJIA	Juvenile (idiopathic) arthritis	diagnosis	diagnosis_cd	M08411
potential sJIA	Juvenile (idiopathic) arthritis	diagnosis	diagnosis_cd	M08872
potential sJIA	Juvenile (idiopathic) arthritis	diagnosis	diagnosis_cd	M08442
potential sJIA	Juvenile (idiopathic) arthritis	diagnosis	diagnosis_cd	M08819
potential sJIA	Juvenile (idiopathic) arthritis	diagnosis	diagnosis_cd	M08049
potential sJIA	Juvenile (idiopathic) arthritis	diagnosis	diagnosis_cd	M08879
potential sJIA	Juvenile (idiopathic) arthritis	diagnosis	diagnosis_cd	M08
potential sJIA	Juvenile (idiopathic) arthritis	diagnosis	diagnosis_cd	M08432
potential sJIA	Juvenile (idiopathic) arthritis	diagnosis	diagnosis_cd	M08971
potential sJIA	Juvenile (idiopathic) arthritis	diagnosis	diagnosis_cd	M08441
potential sJIA	Juvenile (idiopathic) arthritis	diagnosis	diagnosis_cd	M08039
potential sJIA	Juvenile (idiopathic) arthritis	diagnosis	diagnosis_cd	M08271
potential sJIA	Juvenile (idiopathic) arthritis	diagnosis	diagnosis_cd	M08951
potential sJIA	Juvenile (idiopathic) arthritis	diagnosis	diagnosis_cd	M08421
potential sJIA	Juvenile (idiopathic) arthritis	diagnosis	diagnosis_cd	M08972
potential sJIA	Juvenile (idiopathic) arthritis	diagnosis	diagnosis_cd	M08219
potential sJIA	Juvenile (idiopathic) arthritis	diagnosis	diagnosis_cd	M0883
potential sJIA	Juvenile (idiopathic) arthritis	diagnosis	diagnosis_cd	M08939
potential sJIA	Juvenile (idiopathic) arthritis	diagnosis	diagnosis_cd	M08231
potential sJIA	Juvenile (idiopathic) arthritis	diagnosis	diagnosis_cd	M080
potential sJIA	Juvenile (idiopathic) arthritis	diagnosis	diagnosis_cd	M08831
potential sJIA	Juvenile (idiopathic) arthritis	diagnosis	diagnosis_cd	M08262
potential sJIA	Juvenile (idiopathic) arthritis	diagnosis	diagnosis_cd	M08452
potential sJIA	Juvenile (idiopathic) arthritis	diagnosis	diagnosis_cd	M08061
potential sJIA	Juvenile (idiopathic) arthritis	diagnosis	diagnosis_cd	M08251
potential sJIA	Juvenile (idiopathic) arthritis	diagnosis	diagnosis_cd	M08261

potential sJIA	Juvenile (idiopathic) arthritis	diagnosis	diagnosis_cd	M084
potential sJIA	Juvenile (idiopathic) arthritis	diagnosis	diagnosis_cd	M08252
potential sJIA	Juvenile (idiopathic) arthritis	diagnosis	diagnosis_cd	M08829
potential sJIA	Juvenile (idiopathic) arthritis	diagnosis	diagnosis_cd	M08942
potential sJIA	Juvenile (idiopathic) arthritis	diagnosis	diagnosis_cd	M0888
potential sJIA	Juvenile (idiopathic) arthritis	diagnosis	diagnosis_cd	M08841
potential sJIA	Juvenile (idiopathic) arthritis	diagnosis	diagnosis_cd	M08812
potential sJIA	Juvenile (idiopathic) arthritis	diagnosis	diagnosis_cd	M089A
potential sJIA	Juvenile (idiopathic) arthritis	diagnosis	diagnosis_cd	M08079
potential sJIA	Juvenile (idiopathic) arthritis	diagnosis	diagnosis_cd	M08919
potential sJIA	Juvenile (idiopathic) arthritis	diagnosis	diagnosis_cd	M08242
potential sJIA	Juvenile (idiopathic) arthritis	diagnosis	diagnosis_cd	M08269
potential sJIA	Juvenile (idiopathic) arthritis	diagnosis	diagnosis_cd	M08811
potential sJIA	Juvenile (idiopathic) arthritis	diagnosis	diagnosis_cd	M08471
potential sJIA	Juvenile (idiopathic) arthritis	diagnosis	diagnosis_cd	M08011
potential sJIA	Juvenile (idiopathic) arthritis	diagnosis	diagnosis_cd	M0848
potential sJIA	Juvenile (idiopathic) arthritis	diagnosis	diagnosis_cd	M08029
potential sJIA	Juvenile (idiopathic) arthritis	diagnosis	diagnosis_cd	M0820
potential sJIA	Juvenile (idiopathic) arthritis	diagnosis	diagnosis_cd	M08439
potential sJIA	Juvenile (idiopathic) arthritis	diagnosis	diagnosis_cd	M08832
potential sJIA	Juvenile (idiopathic) arthritis	diagnosis	diagnosis_cd	M08249
potential sJIA	Juvenile (idiopathic) arthritis	diagnosis	diagnosis_cd	M0800
potential sJIA	Juvenile (idiopathic) arthritis	diagnosis	diagnosis_cd	M0829
potential sJIA	Juvenile (idiopathic) arthritis	diagnosis	diagnosis_cd	M08052
potential sJIA	Juvenile (idiopathic) arthritis	diagnosis	diagnosis_cd	M08851
potential sJIA	Juvenile (idiopathic) arthritis	diagnosis	diagnosis_cd	M08431
potential sJIA	Juvenile (idiopathic) arthritis	diagnosis	diagnosis_cd	M08849
potential sJIA	Juvenile (idiopathic) arthritis	diagnosis	diagnosis_cd	M08221
potential sJIA	Juvenile (idiopathic) arthritis	diagnosis	diagnosis_cd	M083
potential sJIA	Juvenile (idiopathic) arthritis	diagnosis	diagnosis_cd	M08962
potential sJIA	Juvenile (idiopathic) arthritis	diagnosis	diagnosis_cd	M08031
potential sJIA	Juvenile (idiopathic) arthritis	diagnosis	diagnosis_cd	M089
potential sJIA	Juvenile (idiopathic) arthritis	diagnosis	diagnosis_cd	M0802
potential sJIA	Juvenile (idiopathic) arthritis	diagnosis	diagnosis_cd	M08821
potential sJIA	Juvenile (idiopathic) arthritis	diagnosis	diagnosis_cd	M082
potential sJIA	Juvenile (idiopathic) arthritis	diagnosis	diagnosis_cd	M08222
potential sJIA	Juvenile (idiopathic) arthritis	diagnosis	diagnosis_cd	M08449
potential chest opacity	abnormal imaging finding of the lung	diagnosis	diagnosis_cd	R918
potential chest opacity	abnormal findings other organs (contains chest)	diagnosis	diagnosis_cd	R938
potential chest opacity	abnormal findings other organs (contains chest)	diagnosis	diagnosis_cd	R93811
potential chest opacity	abnormal findings other organs (contains chest)	diagnosis	diagnosis_cd	R93812
potential chest opacity	abnormal findings other organs (contains chest)	diagnosis	diagnosis_cd	R93813
potential chest opacity	abnormal findings other organs (contains chest)	diagnosis	diagnosis_cd	R93819
potential chest opacity	abnormal findings other organs (contains chest)	diagnosis	diagnosis_cd	R9389
potential chest opacity	viral pneumonia	diagnosis	diagnosis_cd	J12
potential chest opacity	viral pneumonia	diagnosis	diagnosis_cd	J120
potential chest opacity	viral pneumonia	diagnosis	diagnosis_cd	J121
potential chest opacity	viral pneumonia	diagnosis	diagnosis_cd	J122
potential chest opacity	viral pneumonia	diagnosis	diagnosis_cd	J123
potential chest opacity	viral pneumonia	diagnosis	diagnosis_cd	J128
potential chest opacity	viral pneumonia	diagnosis	diagnosis_cd	J1281
potential chest opacity	viral pneumonia	diagnosis	diagnosis_cd	J1282
potential chest opacity	viral pneumonia	diagnosis	diagnosis_cd	J1289
potential chest opacity	viral pneumonia	diagnosis	diagnosis_cd	J129
potential chest opacity	other pneumonia	diagnosis	diagnosis_cd	J13
potential chest opacity	other pneumonia	diagnosis	diagnosis_cd	J14
potential chest opacity	other pneumonia	diagnosis	diagnosis_cd	J1426
potential chest opacity	other pneumonia	diagnosis	diagnosis_cd	J15
potential chest opacity	other pneumonia	diagnosis	diagnosis_cd	J150
potential chest opacity	other pneumonia	diagnosis	diagnosis_cd	J151
potential chest opacity	other pneumonia	diagnosis	diagnosis_cd	J152
potential chest opacity	other pneumonia	diagnosis	diagnosis_cd	J1520
potential chest opacity	other pneumonia	diagnosis	diagnosis_cd	J1521
potential chest opacity	other pneumonia	diagnosis	diagnosis_cd	J15211
potential chest opacity	other pneumonia	diagnosis	diagnosis_cd	J15212
potential chest opacity	other pneumonia	diagnosis	diagnosis_cd	J1529
potential chest opacity	other pneumonia	diagnosis	diagnosis_cd	J153
potential chest opacity	other pneumonia	diagnosis	diagnosis_cd	J154
potential chest opacity	other pneumonia	diagnosis	diagnosis_cd	J155
potential chest opacity	other pneumonia	diagnosis	diagnosis_cd	J156
potential chest opacity	other pneumonia	diagnosis	diagnosis_cd	J157
potential chest opacity	other pneumonia	diagnosis	diagnosis_cd	J158
potential chest opacity	other pneumonia	diagnosis	diagnosis_cd	J159
potential chest opacity	other pneumonia	diagnosis	diagnosis_cd	J16
potential chest opacity	other pneumonia	diagnosis	diagnosis_cd	J160
potential chest opacity	other pneumonia	diagnosis	diagnosis_cd	J168
potential chest opacity	other pneumonia	diagnosis	diagnosis_cd	J18
potential chest opacity	other pneumonia	diagnosis	diagnosis_cd	J180
potential chest opacity	other pneumonia	diagnosis	diagnosis_cd	J181
potential chest opacity	other pneumonia	diagnosis	diagnosis_cd	J182
potential chest opacity	other pneumonia	diagnosis	diagnosis_cd	J188
potential chest opacity	other pneumonia	diagnosis	diagnosis_cd	J189
glucocorticoids	betamethasone	patient_report	ndc	168003315
glucocorticoids	betamethasone	patient_report	ndc	168003346
glucocorticoids	betamethasone	patient_report	ndc	168004015
glucocorticoids	betamethasone	patient_report	ndc	168004160
glucocorticoids	betamethasone	patient_report	ndc	168005515
glucocorticoids	betamethasone	patient_report	ndc	168025815
glucocorticoids	betamethasone	patient_report	ndc	472038015
glucocorticoids	betamethasone	patient_report	ndc	472038145
glucocorticoids	betamethasone	patient_report	ndc	517072001
glucocorticoids	betamethasone	patient_report	ndc	16714049601
glucocorticoids	betamethasone	patient_report	ndc	51672127401
glucocorticoids	betamethasone	patient_report	ndc	51672404801
glucocorticoids	betamethasone	patient_report	ndc	51672404806
glucocorticoids	betamethasone	patient_report	ndc	51754506001
glucocorticoids	betamethasone	patient_report	ndc	63323028635
glucocorticoids	betamethasone	patient_report	ndc	63323046837
glucocorticoids	betamethasone	patient_report	ndc	63323046837

glucocorticoids	betamethasone	patient_report ndc	68462029817
glucocorticoids	betamethasone	patient_report ndc	68462029855
glucocorticoids	dexamethasone	patient_report ndc	6394132
glucocorticoids	dexamethasone	patient_report ndc	23334807
glucocorticoids	dexamethasone	patient_report ndc	54418425
glucocorticoids	dexamethasone	patient_report ndc	54817425
glucocorticoids	dexamethasone	patient_report ndc	54817525
glucocorticoids	dexamethasone	patient_report ndc	54817625
glucocorticoids	dexamethasone	patient_report ndc	54818325
glucocorticoids	dexamethasone	patient_report ndc	65064835
glucocorticoids	dexamethasone	patient_report ndc	65853302
glucocorticoids	dexamethasone	patient_report ndc	78087601
glucocorticoids	dexamethasone	patient_report ndc	641036721
glucocorticoids	dexamethasone	patient_report ndc	641036725
glucocorticoids	dexamethasone	patient_report ndc	641614501
glucocorticoids	dexamethasone	patient_report ndc	641614525
glucocorticoids	dexamethasone	patient_report ndc	904530661
glucocorticoids	dexamethasone	patient_report ndc	24208029505
glucocorticoids	dexamethasone	patient_report ndc	24208072002
glucocorticoids	dexamethasone	patient_report ndc	43598032675
glucocorticoids	dexamethasone	patient_report ndc	55150023701
glucocorticoids	dexamethasone	patient_report ndc	60219204301
glucocorticoids	dexamethasone	patient_report ndc	60219204401
glucocorticoids	dexamethasone	patient_report ndc	61314063136
glucocorticoids	dexamethasone	patient_report ndc	61314064705
glucocorticoids	dexamethasone	patient_report ndc	61314064725
glucocorticoids	dexamethasone	patient_report ndc	63323016501
glucocorticoids	dexamethasone	patient_report ndc	63323016505
glucocorticoids	dexamethasone	patient_report ndc	63323016526
glucocorticoids	dexamethasone	patient_report ndc	63323050601
glucocorticoids	dexamethasone	patient_report ndc	63323051610
glucocorticoids	dexamethasone	patient_report ndc	66993073002
glucocorticoids	dexamethasone	patient_report ndc	67457042312
glucocorticoids	dexamethasone	patient_report ndc	68462058576
glucocorticoids	dexamethasone	patient_report ndc	70069002125
glucocorticoids	dexamethasone	patient_report ndc	76045010320
glucocorticoids	dexamethasone	patient_report ndc	76045010610
glucocorticoids	dexamethasone	patient_report ndc	76045010910
glucocorticoids	dexamethasone_unconventional_ndc	patient_report ndc	88888300520
glucocorticoids	fludrocortisone	patient_report ndc	115703301
glucocorticoids	fludrocortisone	patient_report ndc	555099702
glucocorticoids	fludrocortisone	patient_report ndc	42291076401
glucocorticoids	fludrocortisone	patient_report ndc	68084028801
glucocorticoids	fludrocortisone	patient_report ndc	68084028811
glucocorticoids	fludrocortisone	patient_report ndc	70954025230
glucocorticoids	hydrocortisone	patient_report ndc	9001104
glucocorticoids	hydrocortisone	patient_report ndc	9082501
glucocorticoids	hydrocortisone	patient_report ndc	37682210
glucocorticoids	hydrocortisone	patient_report ndc	54455015
glucocorticoids	hydrocortisone	patient_report ndc	65853110
glucocorticoids	hydrocortisone	patient_report ndc	143125401
glucocorticoids	hydrocortisone	patient_report ndc	143970001
glucocorticoids	hydrocortisone	patient_report ndc	168001531
glucocorticoids	hydrocortisone	patient_report ndc	168002031
glucocorticoids	hydrocortisone	patient_report ndc	168008016
glucocorticoids	hydrocortisone	patient_report ndc	168015431
glucocorticoids	hydrocortisone	patient_report ndc	472034356
glucocorticoids	hydrocortisone	patient_report ndc	603390021
glucocorticoids	hydrocortisone	patient_report ndc	603390121
glucocorticoids	hydrocortisone	patient_report ndc	713066815
glucocorticoids	hydrocortisone	patient_report ndc	10631040701
glucocorticoids	hydrocortisone	patient_report ndc	16477020112
glucocorticoids	hydrocortisone	patient_report ndc	24208063110
glucocorticoids	hydrocortisone	patient_report ndc	24208063562
glucocorticoids	hydrocortisone	patient_report ndc	45802001405
glucocorticoids	hydrocortisone	patient_report ndc	45802043803
glucocorticoids	hydrocortisone	patient_report ndc	45802046564
glucocorticoids	hydrocortisone	patient_report ndc	45802047265
glucocorticoids	hydrocortisone	patient_report ndc	49908015030
glucocorticoids	hydrocortisone	patient_report ndc	50383090110
glucocorticoids	hydrocortisone	patient_report ndc	51672300302
glucocorticoids	hydrocortisone	patient_report ndc	51672300701
glucocorticoids	hydrocortisone	patient_report ndc	58468008001
glucocorticoids	hydrocortisone	patient_report ndc	59762007301
glucocorticoids	hydrocortisone	patient_report ndc	59762007401
glucocorticoids	hydrocortisone	patient_report ndc	61269034356
glucocorticoids	hydrocortisone	patient_report ndc	61314064175
glucocorticoids	hydrocortisone	patient_report ndc	61314064610
glucocorticoids	hydrocortisone	patient_report ndc	61570003150
glucocorticoids	hydrocortisone	patient_report ndc	61570003410
glucocorticoids	hydrocortisone	patient_report ndc	61570003675
glucocorticoids	hydrocortisone	patient_report ndc	61570003875
glucocorticoids	hydrocortisone	patient_report ndc	62559013807
glucocorticoids	hydrocortisone	patient_report ndc	62559043001
glucocorticoids	hydrocortisone	patient_report ndc	62559043130
glucocorticoids	hydrocortisone	patient_report ndc	64380097106
glucocorticoids	hydrocortisone	patient_report ndc	64980030130
glucocorticoids	hydrocortisone	patient_report ndc	64980044801
glucocorticoids	hydrocortisone	patient_report ndc	65628001001
glucocorticoids	hydrocortisone	patient_report ndc	67457045450
glucocorticoids	hydrocortisone	patient_report ndc	67887010030
glucocorticoids	hydrocortisone	patient_report ndc	68084046911
glucocorticoids	hydrocortisone	patient_report ndc	68084093025
glucocorticoids	hydrocortisone	patient_report ndc	68220014015
glucocorticoids	hydrocortisone	patient_report ndc	68220014210
glucocorticoids	hydrocortisone	patient_report ndc	68220014410
glucocorticoids	hydrocortisone	patient_report ndc	68462018117
glucocorticoids	hydrocortisone	patient_report ndc	68462057565
glucocorticoids	hydrocortisone	patient_report ndc	69315030230

glucocorticoids	hydrocortisone	patient_report ndc	69315031228
glucocorticoids	hydrocortisone	patient_report ndc	70403092230
glucocorticoids	hydrocortisone	patient_report ndc	71321060312
glucocorticoids	hydrocortisone	patient_report ndc	71399012001
glucocorticoids	hydrocortisone	patient_report ndc	71399018001
glucocorticoids	hydrocortisone	patient_report ndc	72789006201
glucocorticoids	hydrocortisone	patient_report ndc	72789009401
glucocorticoids	hydrocortisone	patient_report ndc	72789009550
glucocorticoids	hydrocortisone	patient_report ndc	75834014724
glucocorticoids	hydrocortisone	patient_report ndc	79503020230
glucocorticoids	methylprednisolone	patient_report ndc	9001820
glucocorticoids	methylprednisolone	patient_report ndc	9003928
glucocorticoids	methylprednisolone	patient_report ndc	9003930
glucocorticoids	methylprednisolone	patient_report ndc	9004722
glucocorticoids	methylprednisolone	patient_report ndc	9005604
glucocorticoids	methylprednisolone	patient_report ndc	9030602
glucocorticoids	methylprednisolone	patient_report ndc	9069801
glucocorticoids	methylprednisolone	patient_report ndc	9075801
glucocorticoids	methylprednisolone	patient_report ndc	9307301
glucocorticoids	methylprednisolone	patient_report ndc	9307303
glucocorticoids	methylprednisolone	patient_report ndc	9347501
glucocorticoids	methylprednisolone	patient_report ndc	9347503
glucocorticoids	methylprednisolone	patient_report ndc	603459315
glucocorticoids	methylprednisolone	patient_report ndc	703003101
glucocorticoids	methylprednisolone	patient_report ndc	703003104
glucocorticoids	methylprednisolone	patient_report ndc	703005101
glucocorticoids	methylprednisolone	patient_report ndc	781502201
glucocorticoids	methylprednisolone	patient_report ndc	781502207
glucocorticoids	methylprednisolone	patient_report ndc	42806040021
glucocorticoids	methylprednisolone	patient_report ndc	43598012725
glucocorticoids	methylprednisolone	patient_report ndc	59746000106
glucocorticoids	methylprednisolone	patient_report ndc	63323025503
glucocorticoids	methylprednisolone	patient_report ndc	63323025803
glucocorticoids	methylprednisolone	patient_report ndc	63323026530
glucocorticoids	methylprednisolone	patient_report ndc	68084014901
glucocorticoids	methylprednisolone	patient_report ndc	68382091601
glucocorticoids	methylprednisolone	patient_report ndc	68382091818
glucocorticoids	methylprednisolone	patient_report ndc	68788459302
glucocorticoids	methylprednisolone	patient_report ndc	70121157301
glucocorticoids	methylprednisolone	patient_report ndc	70121157305
glucocorticoids	methylprednisolone	patient_report ndc	70121157401
glucocorticoids	methylprednisolone	patient_report ndc	70121157405
glucocorticoids	methylprednisolone	patient_report ndc	71283063402
glucocorticoids	methylprednisolone	patient_report ndc	72647033101
glucocorticoids	methylprednisolone	patient_report ndc	72647033104
glucocorticoids	prednisolone	patient_report ndc	121075908
glucocorticoids	prednisolone	patient_report ndc	603156758
glucocorticoids	prednisolone	patient_report ndc	24208031705
glucocorticoids	prednisolone	patient_report ndc	24208071510
glucocorticoids	prednisolone	patient_report ndc	50383004224
glucocorticoids	prednisolone	patient_report ndc	50383004248
glucocorticoids	prednisolone	patient_report ndc	60432021208
glucocorticoids	prednisolone	patient_report ndc	60758011905
glucocorticoids	prednisolone	patient_report ndc	61314029705
glucocorticoids	prednisolone	patient_report ndc	61314063705
glucocorticoids	prednisolone	patient_report ndc	61314063710
glucocorticoids	prednisolone	patient_report ndc	61314063715
glucocorticoids	prednisone	patient_report ndc	54001720
glucocorticoids	prednisone	patient_report ndc	54001729
glucocorticoids	prednisone	patient_report ndc	54001820
glucocorticoids	prednisone	patient_report ndc	54001825
glucocorticoids	prednisone	patient_report ndc	54001920
glucocorticoids	prednisone	patient_report ndc	54001925
glucocorticoids	prednisone	patient_report ndc	54872425
glucocorticoids	prednisone	patient_report ndc	54873925
glucocorticoids	prednisone	patient_report ndc	54874025
glucocorticoids	prednisone	patient_report ndc	603533621
glucocorticoids	prednisone	patient_report ndc	603533832
glucocorticoids	prednisone	patient_report ndc	603533921
glucocorticoids	prednisone	patient_report ndc	904692361
glucocorticoids	prednisone	patient_report ndc	6373958810
glucocorticoids	prednisone	patient_report ndc	59746017206
glucocorticoids	prednisone	patient_report ndc	60687012201
glucocorticoids	prednisone	patient_report ndc	60687013401
glucocorticoids	prednisone	patient_report ndc	60687014501
glucocorticoids	prednisone	patient_report ndc	70882011730
glucocorticoids	prednisone	patient_report ndc	70954005610
glucocorticoids	prednisone	patient_report ndc	70954005710
glucocorticoids	prednisone	patient_report ndc	70954005820
glucocorticoids	prednisone	patient_report ndc	70954005910
glucocorticoids	prednisone	patient_report ndc	70954005920
glucocorticoids	prednisone	patient_report ndc	70954005930
glucocorticoids	prednisone	patient_report ndc	70954006030
glucocorticoids	prednisone	patient_report ndc	70954006110
glucocorticoids	triamcinolone	patient_report ndc	3029305
glucocorticoids	triamcinolone	patient_report ndc	65054301
glucocorticoids	triamcinolone	patient_report ndc	168000415
glucocorticoids	triamcinolone	patient_report ndc	168000480
glucocorticoids	triamcinolone	patient_report ndc	168000615
glucocorticoids	triamcinolone	patient_report ndc	168008115
glucocorticoids	triamcinolone	patient_report ndc	703024101
glucocorticoids	triamcinolone	patient_report ndc	713022815
glucocorticoids	triamcinolone	patient_report ndc	713022880
glucocorticoids	triamcinolone	patient_report ndc	713065540
glucocorticoids	triamcinolone	patient_report ndc	41167058003
glucocorticoids	triamcinolone	patient_report ndc	41167580003
glucocorticoids	triamcinolone	patient_report ndc	45802005436
glucocorticoids	triamcinolone	patient_report ndc	45802006405
glucocorticoids	triamcinolone	patient_report ndc	45802006435

glucocorticoids	triamcinolone	patient_report ndc	45802006436
glucocorticoids	triamcinolone	patient_report ndc	45802010901
glucocorticoids	triamcinolone	patient_report ndc	51672126705
glucocorticoids	triamcinolone	patient_report ndc	51672127201
glucocorticoids	triamcinolone	patient_report ndc	51672128408
glucocorticoids	triamcinolone	patient_report ndc	52565001480
glucocorticoids	triamcinolone	patient_report ndc	52565005615
glucocorticoids	triamcinolone	patient_report ndc	61748022060
glucocorticoids	triamcinolone	patient_report ndc	64980032005
glucocorticoids	triamcinolone	patient_report ndc	67877025115
glucocorticoids	triamcinolone	patient_report ndc	67877025145
glucocorticoids	triamcinolone	patient_report ndc	67877025180
glucocorticoids	triamcinolone	patient_report ndc	67877031780
glucocorticoids	triamcinolone	patient_report ndc	67877031815
glucocorticoids	triamcinolone	patient_report ndc	68462013135
glucocorticoids	triamcinolone	patient_report ndc	68462013159
glucocorticoids	triamcinolone	patient_report ndc	68462031417
glucocorticoids	triamcinolone	patient_report ndc	68462079817
glucocorticoids	triamcinolone	patient_report ndc	68462079917
glucocorticoids	triamcinolone	patient_report ndc	70121104902
glucocorticoids	triamcinolone	patient_report ndc	70121104905
glucocorticoids	triamcinolone	patient_report ndc	70121116801
glucocorticoids	triamcinolone	patient_report ndc	70121116901
glucocorticoids	triamcinolone	patient_report ndc	70677001301
glucocorticoids	triamcinolone	patient_report ndc	70752013005
glucocorticoids	triamcinolone	patient_report ndc	70756081115
glucocorticoids	triamcinolone	patient_report ndc	71085000930
glucocorticoids	triamcinolone	patient_report ndc	71384051001
glucocorticoids	triamcinolone	patient_report ndc	72603010801
glucocorticoids	triamcinolone	patient_report ndc	74157090190
glucocorticoids	triamcinolone	patient_report ndc	76420020315
blockade_IL6	sarilumab	patient_report ndc	24591001
blockade_IL6	siltuximab_unknown_ndc	patient_report ndc	57894042101
blockade_IL6	tocilizumab	patient_report ndc	50242013501
blockade_IL6	tocilizumab	patient_report ndc	50242013601
blockade_IL6	tocilizumab	patient_report ndc	50242013701
blockade_IL6	tocilizumab	patient_report ndc	50242013801
blockade_IL6	tocilizumab	patient_report ndc	50242014301
blockade_IL6	tocilizumab_unconventional_ndc	patient_report ndc	88888001765
blockade_IL6	tocilizumab_unconventional_ndc	patient_report ndc	88888300113
blockade_IL1	canakinumab	patient_report ndc	78073461
blockade_IL1	anakinra_unknown_ndc	patient_report ndc	55513017707
blockade_IL1	anakinra_unknown_ndc	patient_report ndc	66658023401
blockade_IL1	anakinra	patient_report ndc	66658023407
blockade_IL1	anakinra	patient_report ndc	66658023428
blockade_jak_tyk	baricitinib	patient_report ndc	2418230
blockade_jak_tyk	baricitinib	patient_report ndc	2473230
blockade_jak_tyk	tofacitinib	patient_report ndc	69100101
blockade_jak_tyk	tofacitinib	patient_report ndc	69100201
blockade_jak_tyk	upadacitinub	patient_report ndc	74230630
blockade_jak_tyk	ruxolitinib	patient_report ndc	50881001060
blockade_jak_tyk	ruxolitinib	patient_report ndc	50881002560
blockade_jak_tyk	pacritinib placebo	patient_report ndc	72482010012
blockade_jak_tyk	baricitinib_unconventional_ndc	patient_report ndc	88888001118
blockade_TNF	Adalimumab	patient_report ndc	74433902
blockade_TNF	certolizumab	patient_report ndc	50474070062
blockade_TNF	certolizumab	patient_report ndc	50474071079
blockade_TNF	etanercept	patient_report ndc	58406042534
blockade_TNF	etanercept	patient_report ndc	58406043504
blockade_TNF	etanercept	patient_report ndc	58406044504
blockade_TNF	Golimumab	patient_report ndc	57894007002
blockade_TNF	Golimumab	patient_report ndc	57894035001
blockade_TNF	INFLIXIMAB	patient_report ndc	69080901
blockade_TNF	Infliximab	patient_report ndc	57894003001
blockade_TNF	Infliximab_unconventional_ndc	patient_report ndc	88888001538
blockade_TNF	Infliximab_unconventional_ndc	patient_report ndc	88888100515
blockade_TNF	Infliximab_unconventional_ndc	patient_report ndc	99999044085
blockade_IL12_or_IL23	guselkumab	patient_report ndc	57894064001
blockade_IL12_or_IL23	risankizumab	patient_report ndc	74204202
blockade_IL12_or_IL23	Ustekinumab	patient_report ndc	57894005427
blockade_IL12_or_IL23	Ustekinumab	patient_report ndc	57894006103
blockade_IL12_or_IL23	Ustekinumab_unconventional_ndc	patient_report ndc	88888100477
blockade_IL17	brodalumab	patient_report ndc	187000402
blockade_IL17	ixekizumab	patient_report ndc	2144511
blockade_IL17	secukinumab	patient_report ndc	78063941
blockade_IL2	Basiliximab	patient_report ndc	78033184
blockade_IL2	Basiliximab	patient_report ndc	78039361
blockade_IL2	Basiliximab_unconventional_ndc	patient_report ndc	77777000114
