# Supplementary material for: Guideline-based strategies to identify severe cytokine release syndrome in COVID-19 and cancer immunotherapy using large-scale electronic health records
Source: Front Digit Health. 2026 Feb 17;7:1625889. doi: 10.3389/fdgth.2025.1625889 (PMC12953395; doi:10.3389/fdgth.2025.1625889)
Supplement: Supplementary file 7 [file Supplementaryfile3.docx]

# Supplementary methods

## Feature definitions (data cleaning)

Optum® COVID-19 data is always annotated with two columns, one column with a code, and a column with a text description. In theory one could use both code or text to identify a feature. After parsing the list of all codes and text descriptions from the tables of interest, we devised a strategy adapted to each table:

- For the medications (reported, prescriptions and administered), we first filtered by text for the names of vasopressors, blinatumomab, steroids, and IL-1 or IL-6 blockers. Then we gathered the NDC codes used, and needed to manually check that they existed. We discarded approximately 10% of NDC codes that were invalid, which represented less than 1% of entries within a drug name. However, we retained invalid NDC codes when more than 20% of reports within a drug name were using an incorrect NDC code. These codes started by 88 and seemed to be Optum® COVID-19 data internal codes.
- For the procedures, every entry was annotated with a code but only 50% with a text description.

We therefore used the following **pipeline for searching procedures codes**:

1/ We did not search procedures based on their description because it was highly heterogeneous, and rather **started with a list of codes of interest manually defined** (excluding the REV code type). Note, we observed that descriptions are most of the time associated with a code (as opposed to the lab values where the codes were omitted 50% of the time).

2/ After requesting procedures based on their code, we **post-filter whether the descriptions match the intended meaning**, across the diversity of description text. We discard manually all descriptions that do not match the intended feature.

- For the lab values, we noted that every entry had a name but a significant amount did not have a code.

We used the following **pipeline for the selection of “cleanly annotated” lab values**.

1/ We **filter lab values by patterns in their test_name** (and not their code), since we saw that there is always a test_name for all the labs of interest we have tested.

2/ We sorted the combinations of *test_code* (present or absent), *test name* (it was always present for the tested lab values), and *result_unit* and calculated how often they were used, within one feature name. We established that a threshold of “0.05%”, i.e., **only annotations that appear in at least 0.05% of reported measures for a lab value**, consistently isolates only very few reporting/reporting methods and describes the large majority of the data.

The explicit list of filtering criteria to assign a feature to all relevant codes in Optum® COVID-19 data is given in the supplementary files, with description of the table, columns and associated values or units.

## Details on filtering ventilation codes

For procedure codes, mainly related to ventilation, we needed to manually identify codes related to different CRS grades. There are three main types of ventilation:

1/ **the high flow cannula**, which is not consistently reported and does not provide a strong ventilation support, and which is not used for grade 4 CRS but qualifies for grade 3 CRS.

2/ **continuous ventilation**, most often with positive pressure (CPAP) (i.e. the gas is given with pressure to better exchange gas), which is normally used in defining grade 4 CRS.

3/ **invasive ventilation**, which uses the same devices as continuous positive pressure, but with trachea intubation or tracheostomy, which also defines grade 4 CRS

The list of ventilation codes used related to the grading level is shown in Table S4.

Of note, a first set of CPT procedures have been used for insurance claims (94660, 94002, 94003; the code 1014859 only pools 94002 and 94003 and does not add information). These codes are made for either CPAP or invasive ventilation are not specific except initiation and number of days. Insurances typically track whether 5 full days of ventilation were required, in which case the reimbursement is higher.

The following set of CPT procedures reported the precise ventilation family/type and duration on a day-by-day basis. Assistance codes mean the patient maintains the control of the breathing, while performance codes mean that the machine takes over respiration and refers to invasive ventilation.

A significant problem is that some health centers have used insurance claim codes, while others have used ventilation family codes. Therefore, we included both sets of codes to distinguish between patients with and without ventilation.

## Vasopressors

We searched the use of vasopressor by first applying a text search on drug names with *“vasop|phenylephrine|norepinephrine|epinephrine|dopamine|dobutamine|milrinone|desmopressin|terlipressin|lypressin|catecholamine|phenylephrine|apomorphine|bromocriptine|cabergoline|rotigotine|ropinirole|pramipexole|pergolide|opicapone|entacapone|tolcapone|amantadine|Monoamine oxidase b inhibitor|levodopa”*, we then pooled all NDC codes used with the found test descriptions, then manually checked the existence of these NDC codes and reported them in the Supplementary Files.
