# Supplementary material for: Guideline-based strategies to identify severe cytokine release syndrome in COVID-19 and cancer immunotherapy using large-scale electronic health records
Source: Front Digit Health. 2026 Feb 17;7:1625889. doi: 10.3389/fdgth.2025.1625889 (PMC12953395; doi:10.3389/fdgth.2025.1625889)
Supplement: Supplementary file 8 [file Image1.pdf]

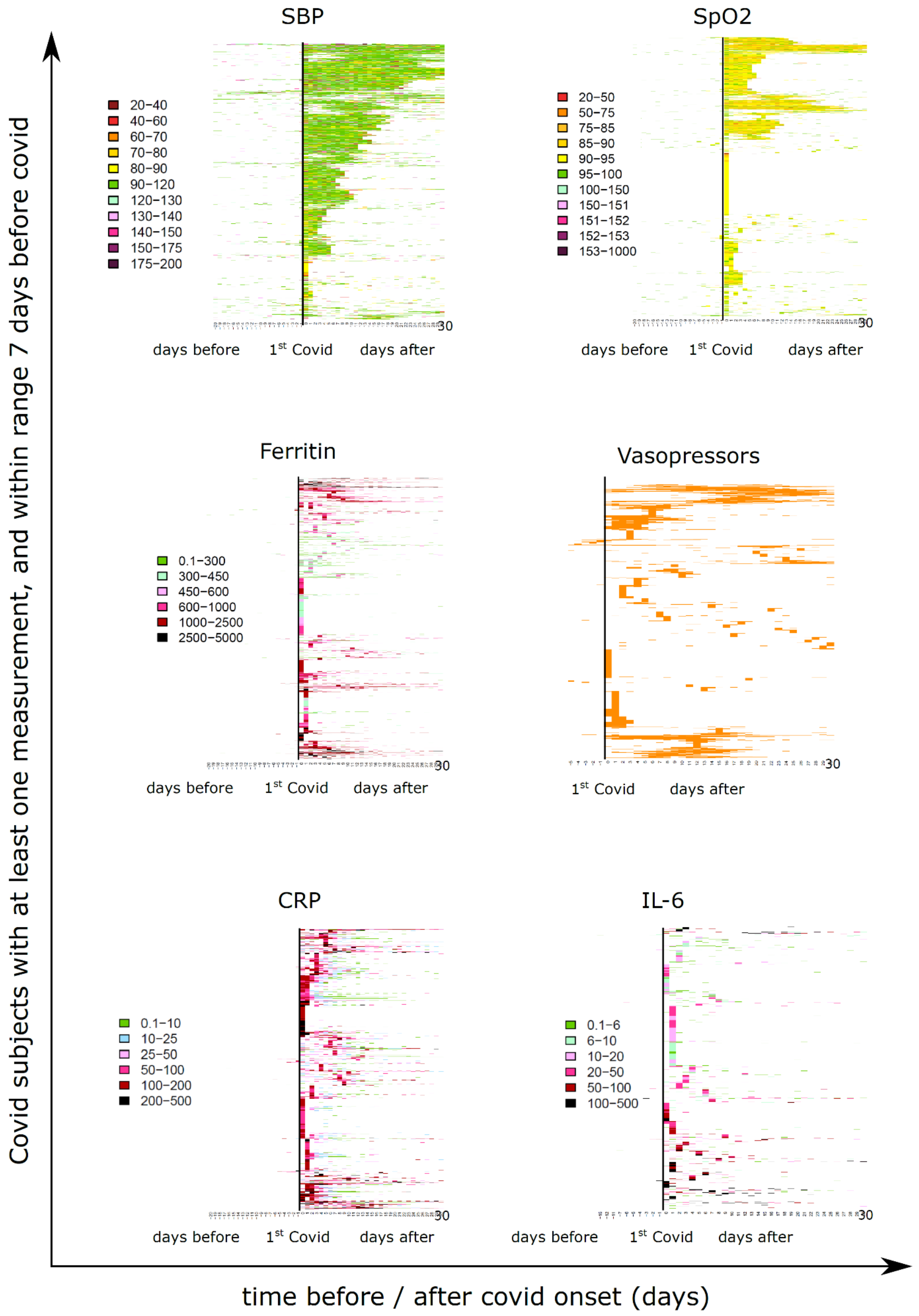

Supplementary Figure 1: Heterogeneity of patient trajectories and reporting periods in the COVID-19 dataset. Selected lab values relevant to inflammation and CRS grading are shown for each patient over time, indicating their maximum values (CRP, IL-6, Ferritin), minimum values (SBP, SpO2), or vasopressor use per day. Patients are clustered separately for each variable.
