# Supplementary material for: Guideline-based strategies to identify severe cytokine release syndrome in COVID-19 and cancer immunotherapy using large-scale electronic health records
Source: Front Digit Health. 2026 Feb 17;7:1625889. doi: 10.3389/fdgth.2025.1625889 (PMC12953395; doi:10.3389/fdgth.2025.1625889)
Supplement: Supplementary file 9 [file Image2.pdf]

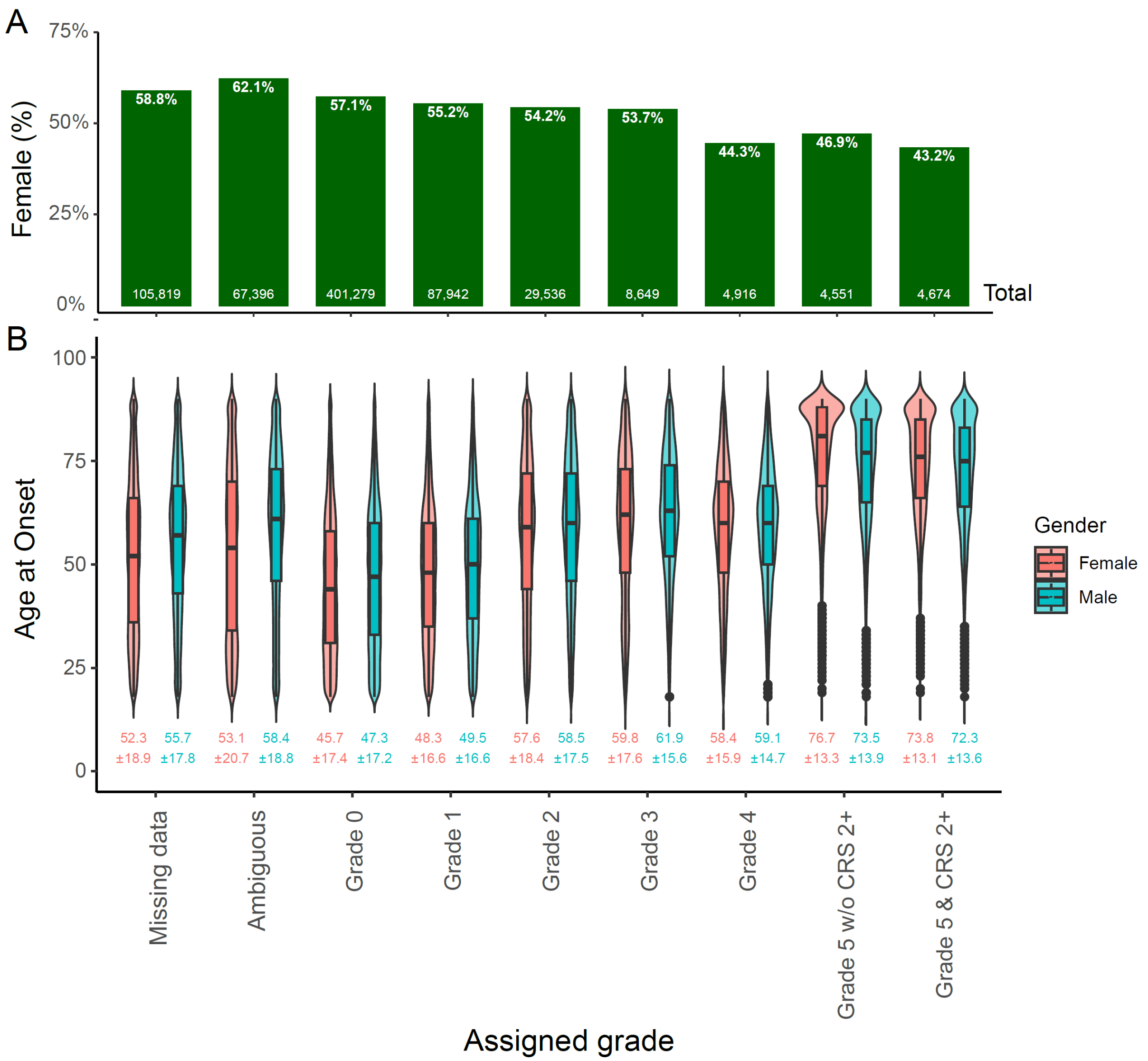

Supplementary Figure 2: Demographics of patients according to their assigned grade by the case identification algorithm. A. Percent of women and total number of subjects within each grade. B. Distribution of ages within men and women of each grade. Patients with grade 0 to 1 are younger at onset compared to patients with grade 2 or more. Deceased patients (Grade 5) are older than all other patients.
